# A Study of Deep Clustering in Spike Sorting

**DOI:** 10.1007/s12021-025-09751-4

**Published:** 2025-10-22

**Authors:** Eugen-Richard Ardelean, Raluca Laura Portase

**Affiliations:** Department of Computer Science, Technical University of Cluj-Napoca, Cluj- Napoca, Romania

**Keywords:** Clustering, Deep clustering, Spike sorting, Neuroscience

## Abstract

Spike sorting is the process of identifying the source neurons for neuronal activity recorded from extracellular electrodes. Traditional spike sorting pipelines separate the process into distinct feature extraction and clustering steps, which may not optimally capture the complex structure of spike data. This study provides a large-scale benchmark of 12 deep clustering algorithms against traditional feature extraction methods combined with K-means clustering for spike sorting. We analyze performance across 95 synthetic datasets with varying cluster counts (2-20) and complexity from the perspective of six performance metrics. Our results demonstrate that a subset of deep clustering algorithms—particularly ACeDeC, DDC, DEC, IDEC and VaDE—significantly outperform traditional methods, especially as dataset complexity increases. These deep clustering approaches effectively learn non-linear representations that better capture the structure of spike data while simultaneously optimizing clustering objectives. This dual optimization produces feature spaces tailored for clustering, combining the two traditionally separate steps of spike sorting. Our findings indicate that deep clustering approaches are most suitable for accurately identifying individual neuronal activity in extracellular recordings, providing guidance for method selection for the increasingly complex modern multi-electrode recordings.

**Supplementary Information:**

The online version contains supplementary material available at https://doi.org/10.1007/s12021-025-09751-4.

## Introduction

### Spike Sorting

Spikes sorting (Bear et al., 2015; Quiroga 2007) is the process of organizing the instances of neuronal action potentials, also known as spikes, into groups depending on the putative neurons. This process is applied to extracellular data where the spikes of multiple neurons in the vicinity is captured by a recording electrode (Carter & Shieh 2015). Therefore, the provenience of each spike is unknown during recording. Spike sorting is often referred to as being analogous (Brown et al., 2001) to the cocktail party problem (Haykin & Chen 2005). The latter requires the isolation of an individual’s speech in complex environment, similarly, spike sorting attempts to extract the words (as in spikes) of a single person (as in neuron) in a complex environment that is riddled with noise and where individuals may speak at once (spike superposition) and at different cadences (different firing rates of neurons). Spike sorting operates on the assumption that each neuron produces spikes of a similar shape, while simultaneously different from the spikes of any other neuron.

In its most traditional form, the spiking sorting process (Quiroga 2007; Buccino et al. 2022; Rey et al. 2015) is separated into four consecutive steps (Bear et al., 2015; Quiroga 2007): filtering of the recording signal, spike detection of the filtered signal, feature extraction of the detected spikes (to reduce dimensionality) and clustering for the assignment of spikes to a specific neuron. The filtering of the recording signals employs a band-pass filter between 300 and 3000 Hz (Rey et al., 2015) to capture the frequency components of spikes. The spike detection step is traditionally a simple amplitude thresholding which imposes a comprise (Zhang et al., 2025) between precision (i.e., proportion of detected events that are true spikes) and recall (i.e., proportion of actual spike events that are detected). Lowering the threshold can increase recall—more true spikes are captured—but at the cost of reduced precision, since more noise events may be falsely detected as spikes. Conversely, raising the threshold improves precision but lowers recall. The optimal balance depends on the specific dataset and application context. In simpler terms, the more spikes are identified the higher is the chance of including noise; however, as many spikes as possible must be extracted. This study focuses on the last two steps of the pipeline, the feature extraction and the clustering. The feature extraction step attempts to identify the most informative features (Ardelean et al., 2023; Mure et al., 2022) for the generation of lower-dimensional working space, while the clustering (Ardelean et al., 2023; Tolas et al., 2023) step attempts to separate clusters in the space obtained by the feature extraction. In this case, the most informative features represent the features that bestow the most separability between clusters (Tolas et al., 2023). Finally, each cluster should represent all instances of activity of a single neuron.

The spike sorting pipeline (Buccino et al., 2022; Rey et al., 2015) has seen a number of iterations during the years, starting from a manual approach (Chung et al., 2017) where spikes were separated and assigned by a researcher based on simple characteristics such as amplitude and width (Meister et al., 1994). One such discriminatory feature found was the peak-to-trough ratio (Ebbesen et al., 2016) which allowed researchers to distinguish between the spikes of inhibitory (narrow spikes) and excitatory (wide spikes) neurons. More complex probabilistic models which could process a low number of electrodes were created that were able to make use of the entire spike waveform (Pouzat et al. 2002). Later, more complex algorithms were employed principal components (Litke et al. 2004), the wavelet transform (Hulata et al. 2002) and various combinations of them to project the high-dimensional space of spikes into a low-dimensional space.

Due to the recent advances in recording hardware (Steinmetz et al., 2021; Jun et al., 2017), such methods are rapidly turning intractable. The number of neurons captured in recordings has been increasing exponentially since the 1950 s (Stevenson & Kording 2011) and now with the development of multi-array silicon probes (Steinmetz et al., 2021; Jun et al., 2017), thousands of neurons can be captured in a single recording. Depending on the approach, online or offline, different variants can be used. In offline spike sorting the use of more sophisticated algorithms is allowed by the lack of a time constraint, while in online spike sorting it must be done during the recording, and a faster approach is required to abide by the time constraints.

Lately, template matching has been increasingly utilized as an alternative for the spike detection and feature extraction steps due to its performance and computational efficiency (Pachitariu et al., 2016), as it is usually applied to only a subset of the dataset. One such method that focuses on the Wavelet Transform for detection and template matching is M-Sorter (Yuan et al., 2012). It detects spikes from the band-pass filtered spike waveforms by computing the correlation of wavelet coefficients, templates are generated through the use of K-Means, and are spikes are matched to the closest templates. Another pipeline that employs template matching and K-Means is KiloSort (Pachitariu et al., 2016; Pachitariu et al., 2024). Kilosort creates spike templates through mathematical models that are then used to initialize a modified K-Means. Computational efficiency is the main advantage of KiloSort; however, it also allows the possibility of human intervention as a post-processing step.

In this work, we endeavor in the pursuit of identifying the suitability of deep clustering algorithms for spike sorting. Although, many feature extraction and clustering algorithms have been employed in the task of spike sorting, no gold standard (Quiroga 2007; Rey et al., 2015; Pedreira et al., 2012; Estivill-Castro 2002) has been yet found as the performance of each algorithm is dependent upon the specific characteristics of the data. Here, we systematically evaluate an array of deep clustering algorithms to determine which are most effective for spike sorting, aiming to establish practical performance baselines.

### Deep Clustering Algorithms

Deep clustering algorithms (Min et al., 2018; Zhou et al., 2024; Lu et al., 2024; Aljalbout et al., 2018; Nutakki et al., 2019; Ren et al., 2025; Wei et al., 2024) are neural network approaches to clustering based on autoencoders (Lopez et al., 2019; Baldi 2012). Traditionally, autoencoders are composed of two inter-linked parts: an encoder and a decoder. Their task is to compress the input data into a latent representation, usually lower-dimensional, and reconstruct the input data at the output. Autoencoders have been applied for many different applications such as feature extraction, dimensionality reduction, generative modelling and anomality detection. Autoencoders have been also been demonstrated to be an adequate approach in the feature extraction of image datasets (Hinton & Salakhutdinov 2006; Wang et al., 2014; Wang et al., 2016), such as MNIST. Due to inherent non-linearity of autoencoders from the activation functions, autoencoders are a suitable approach for the task of spike sorting (Radmanesh et al., 2022).

Traditional clustering methods have been shown to struggle with high-dimensional complex data. Deep clustering algorithms (Min et al., 2018; Zhou et al., 2024; Lu et al., 2024; Aljalbout et al., 2018; Nutakki et al., 2019; Ren et al., 2025; Wei et al., 2024) have been proposed a solution for this issue and have been demonstrated to have a high performance on image datasets (Moradi Fard et al. 2020; Guo et al., 2017; Leiber et al., 2022, 2021; Miklautz et al., 2021; Song et al., 2013; Mautz et al., 2019; Mautz et al. 2020). Most of these methods (Moradi Fard et al., 2020; Guo et al., 2017; Miklautz et al., 2021; Mautz et al., 2019; Yang et al., 2017; Kimura 2018) have been designed with a modified loss function to include both the reconstruction and the clustering as well. A subset of deep clustering methods (Leiber et al., 2022, 2021)have also been designed based on pretraining followed by iterative refinement based on the statistical dip-test (Hartigan & Hartigan 1985)for modality in iterative loops for updating labels (Leiber et al., 2022) or as a postprocessing step of cluster merging (Leiber et al., 2021). Even a tree approach (Mautz et al., 2019) has been designed that uses a joint optimization strategy for clustering. Simpler approaches have also been taken, where a 2-stage approach (Ren et al., 2020) is taken, the autoencoder beings by creating a low-dimensional representation of the input which is then further reduced through the t-SNE algorithm to a 2-dimensional space that is clustered by a density-based approach. Thus, deep learning approaches are strong candidates for spike sorting.

### The Challenges of Spike Sorting

As it was alluded to in the cocktail party problem, spike sorting suffers from an assortment of challenges. Realistically, even if the idea of neural coding would be invalidated, background noise induces variability (Quiroga 2007; Rey et al., 2015) into the shapes of spikes which would still generate clusters. Consequently, feature extraction techniques are an important step in improving the robustness of clustering by removing redundant information. As pointed to above, neurons can have different firing rates (Lewicki 1998; Buzsáki 2006). Within the finite frame of a recording, different firing rates result in a different number of spikes which leads to imbalanced clusters. This happens due to neuronal activity being modulated by entire brain circuits rather than a single neuron. Besides noise, the shape of spikes can be disrupted by phenomena such as electrode drift (Steinmetz et al., 2021). Electrode drift (Steinmetz et al., 2021; Lefebvre et al., 2016) appears due to electrode or tissue movement and manifests as gradual changes in the recorded waveforms. These can lead to more similar spike shapes which result in overlapping clusters. Neuronal action potentials from different neurons may overlap in time resulting in overlapping spikes, also called spike collisions. Neuronal bursting (Bakkum et al., 2014; Ardelean et al., 2023) refers to the phenomenon of individual neurons may produce multiple action potentials with varying waveform shapes and amplitudes in a row in a small amount of time. Finally, the time scale of neuronal activity is of milliseconds, implying that even a brief recording will generate a high volume of data (Bear et al., 2015). From a terminological perspective, single unit activity refers to a cluster that is composed from the spikes of a single neuron, while multiunit activity refers to a “cluster” that is composed of the spikes of multiple neurons (usually more distant from the recording electrode) (Rey et al., 2015).

The aim of feature extraction is to generate a new feature space that is resistant to small changes in spike shape, thus offering separability in clusters. The purpose of clustering is to group the different groups of activity to identify the activity of different neurons. As the spikes of neurons are muddled by the inherent background noise of brain recordings, autoencoder which have been demonstrated a robust ability for denoising may be able to offer a latent representation that is invariant to noise (Lopez et al., 2019). Autoencoders have seen previous use in spike sorting (Radmanesh et al., 2022; Eom et al., 2021)with promising results. Many variants have been developed for the introduction of deep learning into spike sorting (Meyer et al., 2024). Yet, the suitability of deep clustering methods in spike sorting which employ autoencoders has not yet been determined.

The paper is organized in the following manner: Sect. 2 presents the traditional feature extraction methods, clustering methods and performance metrics used in the analysis. Moreover, it provides a description of the proposed methods for spike sorting, and of the datasets. In Sect. 3, a thorough evaluation is made on each method on multiple datasets from the perspective of various performance metrics, simultaneously offering a critical interpretation of the results. Finally, Sect. 4 explores the limitations of our proposal and the conclusions reached.

## Materials and methods

### Feature Extraction

Feature extraction is a key step in the spike sorting pipeline in which spike waveforms are represented through a smaller informative feature space. In spike sorting, for computational reasons, feature extraction attempts to reduce the dimensionality of the original feature space while retaining the information that allows for the discrimination of spikes from different neurons. This implies creating features that are invariant to the noise that differentiates the spikes produced by the same neuron. Techniques for feature extraction methods can be categorized by multiple attributes such as linearity; thus, Principal Component Analysis is a linear convex algorithm while Independent Component Analysis is a linear non-convex approach. Convexity in feature extraction refers to whether the optimization problem involved has a convex objective function and feasible region, ensuring a global optimum. Convex methods guarantee stable, unique solutions, while non-convex methods may have multiple local optima.

#### Linear Feature Extraction Methods

One of the most widely used techniques for feature extraction, in general and in spike sorting (Adamos et al., 2008), is the Principal Component Analysis (PCA) (Mishra et al., 2017). Despite its limitations, PCA has been extensively used in spike sorting over the years (Rey et al., 2015)and it is still is used in modern spike sorting pipelines (Toosi et al., 2021). PCA transforms the input data into a new feature space of orthogonal axes – called principal components – which are derived through eigenvalue decomposition. It can reduce dimensionality by discarding components with low variance. Often by retaining only the first few principal components (Abeles & Goldstein 1977; Glaser et al., 1968), more than 70% of the data variance is captured. However, by retaining variance it is not guaranteed that an optimal space for clustering is created (Quiroga 2007; Rey et al., 2015).

Independent Component Analysis (ICA) (Hyvärinen 2013) is another linear method, generally employed in source separation that has been shown to have applications in spike sorting (Lopes et al., 2013; Tiganj & Mboup 2012). ICA focuses on maximizing independence among the components it can find, rather than variance as PCA does. This unsupervised approach identifies independent sources in the data allowing it to isolate individual instances of neuronal activity and it has been demonstrated to have a high performance in spike sorting (Lopes et al., 2013; Tiganj & Mboup 2012).

#### Non-linear Feature Extraction Methods

Isomap (Tenenbaum et al., 2000) employs Isometric Mapping to create a low-dimensional manifold embedding from the input data while preserving the distances of the original space. It fits into the non-linear category of feature extraction methods, and it has a manifold approach. Isomap builds a graph where nodes are linked to their nearest neighbors and it approximates the geodesic distance (shortest paths in this graph) which are then scaled using Multidimensional Scaling (MDS) (Borg & Groenen 2005). By preserving geodesic distances among data points, Isomap captures the intrinsic relationships in the high-dimensional space. MDS attempts to create a lower-dimensional space that matches the original space (as defined by distances between points) through the minimization of a stress function. As Isomap computes embeddings from a fixed neighborhood graph; thus, projecting new points requires additional approximation techniques such as Nyström extensions (Bengio et al., 2003). However, in our study, all points are embedded jointly, and as such out-of-sample projections are avoided.

Locally Linear Embedding (LLE) (Roweis & Saul 2000) attempts to preserve local structure through the assumption that each neighborhood lies on a linear patch of the manifold. LLE begins by finding the k-nearest neighbors of each data point, then it solves a set of linear equations to compute the best weights to reconstruct each data point based on its neighbors, and finally by solving an eigenvalue problem it finds a low-dimensional embedding that preserves the weights used for the reconstruction.

T-distributed Stochastic Neighbor Embedding (t-SNE) (Zhou et al., 2018) attempts to create a mapping from the high-dimensional data to a lower-dimensional space through pairwise probability similarities using the t-distribution to preserve both local and global structure. T-SNE minimises the Kullback-Leibler divergence between input feature space and the reduced feature space by using the two distributions. Gaussian distributions are used to compute the conditional probabilities that represent the similarities between the points in the original space. T-SNE’s use in clustering pipelines should be interpreted carefully due to the fact that it preserves local neighborhood structure but not global structure, and its stochastic nature can produce different embeddings for each run.

Diffusion Map (DM) (Variable bandwidth diffusion kernels 2016) attempts to identify the data structure through the use of diffusion processes on the manifold. It constructs a graph in which the edges represent the probability of traversal from a point to another in a random walk. It computes the eigenvectors, low dimensional embeddings which preserve diffusion distances, of the normalized graph Laplacian corresponding to various time scales of the diffusion process.

### Traditional Clustering Algorithms

K-Means (MacQueen 1967)is one of the oldest clustering algorithm and has been introduced to spike sorting in 1988 (Salganicoff et al., 1988; Veerabhadrappa et al., 2020). K-Means partitions the input space by assigning data points to their closest*k*centroids which are initialized randomly. Through iterative assignment and optimization of the centroids, clustering is achieved. There are several disadvantages to K-Means: it requires the number of clusters to be known beforehand, it is nondeterministic, it does not handle overlapping clusters or clusters of arbitrary shapes, and it is sensitive to outliers. Since its introduction to spike sorting, K-Means has seen extensive use in this domain (Salganicoff et al., 1988; Veerabhadrappa et al., 2020) and even newly developed pipelines make use of it (Pachitariu 2016; Caro-Martín et al., 2018). Furthermore, it has been shown to still be a strong candidate by placing 3rd out of 25 (Veerabhadrappa et al., 2020) clustering algorithms in a comprehensive analysis of clustering algorithms. K-Means was selected as the baseline clustering method in our traditional spike sorting pipeline because it remains the most widely used approach in this field (Pachitariu et al., 2016; Salganicoff et al., 1988; Veerabhadrappa et al., 2020; Caro-Martín et al., 2018), and enables direct comparison with prior work.

While alternatives such as DBSCAN exist, these present notable drawbacks in the context of our benchmarking. Density-Based Spatial Clustering of Applications with Noise (Ester 1996), better known as DBSCAN, is a density-based approach that also seen use in the domain of spike sorting (Veerabhadrappa et al., 2020). DBSCAN builds clusters by first identifying their cores as zones of high density and expanding them, while low density zones are considered noise. DBSCAN functionality is based upon two highly sensitive parameters, but it does not require knowledge of the number of clusters beforehand, also DBSCAN is able to handle clusters of arbitrary shape, and it is mostly deterministic (excluding border points). DBSCAN is known to struggle with datasets containing clusters of varying densities, and the imbalanced firing rates in spike data can lead to such imbalanced clusters resulting in the misclassification of sparse clusters as noise. Furthermore, DBSCAN has a higher time and space complexity, making it less scalable to large datasets.

### Deep Clustering Algorithms

Traditional clustering algorithms struggle with complex data structures (Min et al., 2018; Zhou et al., 2024; Lu et al., 2024; Aljalbout et al., 2018; Nutakki et al., 2019; Ren et al., 2025; Wei et al., 2024). Deep clustering techniques (27–33) combine representation learning with clustering objectives to enhance performance, often using autoencoders (Lopez et al., 2019; Ardelean et al., 2023). Most of these methods have been tested on the MNIST dataset (Moradi Fard et al., 2020; Guo et al., 2017; Leiber et al., 2022, 2021; Miklautz et al., 2021; Song et al., 2013; Mautz et al., 2019; Mautz et al. 2020), showing a satisfactory performance in clustering high dimensional datasets; thus, proving their potential for complex tasks such as spike sorting. The deep clustering algorithms analyzed here have their code provided by the authors. For consistency of the results, we have used the implementations of these algorithms from clustpy (Leiber et al., 2023), with some modifications to improve performance. A short description of each deep clustering method with its key characteristics can be found in Table S1 in the Supplementary Material.

ACeDeC (Miklautz et al., 2021), introduced in 2021, is a deep clustering approach that separates the latent representation into distinct spaces: a clustering space for cluster-specific information and a shared space for general data variation. ACeDeC measures the importance of each dimension within these spaces. Additionally, the loss function used accounts for the cluster information by minimizing distances to centroids, the shared information by modelling the distance to the mean of the embedded data and for the reconstruction of the autoencoder. By separating the embedded space and using a reformulated loss function, ACeDeC enables the learning of detailed reconstructions and cluster-specific abstractions, and it improves clustering performance. Experiments on various datasets demonstrate ACeDeC’s superior performance compared to existing methods, even DCN (Yang et al., 2017) another deep clustering approach.

AEC (Song et al., 2013), introduced in 2013, is a deep clustering approach that proposes using autoencoders for mapping data to a more suitable space. This method incorporates both data reconstruction and cluster compactness through its proposed loss function, leading to more stable and effective clustering. The model iteratively refines data representation and cluster centres, achieving superior performance compared to conventional approaches like K-means. Experiments on benchmark datasets demonstrate the improved accuracy and normalised mutual information of this auto-encoder-based clustering technique.

DCN (Yang 2017), introduced in 2017, proposes the use of deep neural networks (DNNs) for dimensionality reduction and K-means for the clustering of high-dimensional data. This method learns a ‘clustering-friendly’ latent space by simultaneously optimising data reconstruction, dimensionality reduction, and cluster structure. DCN uses an autoencoder network structure (with a step of greedy layer-wise pre-training (Bengio et al., 2007)) with a K-means clustering objective at the bottleneck layer to avoid trivial solutions, and an alternating stochastic gradient algorithm for optimisation. Experiments on synthetic and real-world datasets demonstrate the effectiveness of DCN in improving clustering performance compared to state-of-the-art methods, particularly in cases with unbalanced clusters. It was shown to outperform other deep clustering approaches, such as DEC (Xie et al., 2016) and simpler approaches that used an autoencoder to reduce dimensionality and a clustering algorithm such as K-Means.

DDC (Ren et al., 2020), introduced in 2020, employs a two-stage approach: first, it uses a deep convolutional autoencoder to learn low-dimensional feature representations, and then applies a new density-based clustering technique. DDC uses a deep autoencoder to learn deep feature representations of data. It adopts t-SNE to further reduce the learned features to a 2-dimensional space while preserving the pairwise similarity of data instances. It develops a novel density-based clustering method that considers both the local structures of clusters and the importance of instances to generate the final clustering results. This method addresses limitations in existing deep clustering algorithms, specifically the need for a pre-defined number of clusters and instability with non-spherical cluster shapes. Experiments demonstrate that DDC achieves state-of-the-art performance, even when the number of clusters is unknown, making it a robust solution for various image clustering tasks. Moreover, DDC was shown to outperform other deep clustering methods, specifically DEC (Xie et al., 2016), IDEC (Guo et al., 2017), DKM (Moradi Fard et al., 2020) and VaDE (Jiang et al., 2017).

DEC (Xie et al., 2016), introduced in 2016, proposes the use of DNNs, specifically an autoencoder, to simultaneously learn feature representations and cluster assignments. It iteratively refines clusters by optimising a clustering objective in a lower-dimensional space. This process involves computing soft assignments and minimising Kullback-Leibler divergence using an auxiliary target distribution to map the autoencoder’s embeddings to cluster centroids. DEC applies a greedy layer-wise pre-training (Bengio et al., 2007) on the autoencoder starting with weights initialized from a normal distribution. The authors demonstrate significant improvements over existing clustering methods on image and text datasets. Furthermore, DEC exhibits robustness to hyperparameter variations, making it practical for real-world applications. The algorithm’s linear complexity enables it to scale effectively to large datasets.

DeepECT (Mautz et al., 2019; Mautz et al., 2020), introduced in 2019, is a deep hierarchical clustering approach that combines the strengths of deep learning and traditional clustering methods. It uses a generic feedforward autoencoder with a clustering layer that builds a cluster tree (without needing the number of clusters specified beforehand) in an embedded space, and both the embedding and the tree are trained simultaneously. DeepECT uses a projection-based optimization strategy that enhances cluster boundaries and preserves orthogonal structural information through a compression loss that penalizes the distance between data points and their assigned node centers. It also includes an extension that utilizes augmentation methods to ignore known invariances within the data. Experimental results demonstrate that DeepECT excels in creating high-quality cluster trees and performs competitively with flat clustering methods. It was shown to outperform other deep clustering approaches, such as IDEC (Guo 2017) and simpler approaches that used an autoencoder to reduce dimensionality and a clustering algorithm such as K-Means.

DipDECK (Leiber et al., 2021), introduced in 2021, is a deep clustering approach that simultaneously learns data representations and estimates the number of clusters present. DipDECK integrates a cluster number estimation within the deep learning process, addressing limitations in scalability and reliance on pre-defined cluster numbers. The algorithm uses an autoencoder to embed data, overestimates the initial cluster count, and then applies Hartigan’s Dip-test to merge structurally similar clusters. Experiments demonstrate that DipDECK achieves competitive clustering results, accurately estimates cluster numbers, and exhibits robustness across various datasets and parameter settings. Moreover, it was compared with other deep clustering methods and shown to outperform them, specifically, DEC (Xie et al., 2016), IDEC (Guo et al., 2017), DCN (Yang et al., 2017) and VaDE (Jiang et al., 2017) on 7 out of 8 datasets.

DipEncoder (Leiber et al., 2022), introduced in 2022, is a deep clustering algorithm that leverages Hartigan’s Dip-test to enforce multimodality in autoencoders. This approach combines an autoencoder with the Dip-test, enabling the creation of embeddings that clearly separate clusters within a dataset. The DipEncoder uses gradients of the Dip-value with respect to both the projection axis and the data itself to improve cluster separation. It uses two loss terms, one to minimize the modality of within separate clusters and another to maximize modality between combinations of clusters. The algorithm updates cluster labels using the Dip-test and requires only the number of clusters as a parameter, offering a parameter-free method for deep clustering. By maximizing multimodality between clusters while ensuring unimodality within individual clusters, the DipEncoder achieves competitive performance compared to state-of-the-art deep clustering methods, specifically, DEC (Xie et al., 2016), IDEC (Guo et al., 2017), DCN (Yang et al., 2017) and DipDECK (Leiber et al., 2021) on 6 out of 10 various datasets, including image, numerical, and text data.

DKM (Moradi Fard et al., 2020), introduced in 2020, is a deep clustering algorithm that jointly learns data representations and performs K-Means clustering. It uses joint optimization through stochastic gradient descent to learn autoencoder-based representations, and it uses a differentiable parametrized softmax instead of argmin for K-Means. It uses a greedy layer-wise pre-training (Bengio et al., 2007)for the autoencoder in one variant and an annealing approach for a second variant. DKM uses a continuous reparameterization of the objective function. Experiments on image and text datasets demonstrate DKM’s superior clustering performance compared to other deep clustering models such as DCN (Yang et al., 2017) and IDEC (Guo et al., 2017). The pretrained variant obtained a slightly higher and more stable performance when compared with the annealing variant.

IDEC (Guo et al., 2017), introduced in 2017, is a deep clustering approach that seeks to simultaneously cluster data and learn meaningful feature representations by integrating an autoencoder with a clustering loss function. This combination allows the algorithm to scatter data points while preserving the local structure of the data. It is stated that preserving this structure is vital for effective deep clustering as clustering losses can sometimes corrupt the feature space, leading to non-representative and meaningless features. IDEC uses an under-complete autoencoder. IDEC uses a stacked denoising autoencoder (with a step of greedy layer-wise pre-training (Bengio et al., 2007)), followed by an under-complete (the latent code is of lower size than the input) autoencoder after initialization to preserve the local structure of the data generating distribution. This constrains the manipulation of the feature space while using a clustering loss to scatter data points. Moreover, IDEC has been shown to outperform its precursor DEC (Xie et al., 2016) and simpler approaches that used an autoencoder to reduce dimensionality and a clustering algorithm such as K-Means.

N2D (McConville et al., 2020), introduced in 2021, is a deep clustering approach that simplifies existing methods by replacing a deep clustering network with manifold learning. N2D uses an autoencoder to create an initial data representation, then employs manifold learning techniques, especially UMAP, to uncover a more cluster-friendly structure. This manifold learning step focuses on preserving local distances while retaining global structure, improving cluster quality. The resulting embedding is then clustered using a shallow algorithm, achieving competitive, and sometimes superior, performance on image and time-series datasets. Experiments demonstrate N2D’s efficiency and effectiveness compared to traditional and state-of-the-art deep clustering methods.

VaDE (Jiang et al., 2017) or Variational Deep Embedding, introduced in 2017, is an unsupervised, generative clustering approach that uses variational autoencoders (VAE). It models data generation by combining a Gaussian Mixture Model (GMM) with a deep neural network (DNN), where the GMM selects a cluster to produce a latent embedding and the DNN decodes this into an observable output. An encoder network is used to infer latent embeddings from observables to maximize the evidence lower bound (ELBO). The method aims to learn suitable representations for clustering tasks and generate realistic samples without supervised training. The experiments presented demonstrate VaDE’s ability to outperform state-of-the-art methods on benchmark datasets.

AutoClustering (Kimura 2018), introduced in 2018, a clustering algorithm based on feed-forward neural networks (FFNN), offering an alternative to methods like Self-Organizing Maps (SOM). This approach employs an encoder-decoder structure and a loss function to map data records to clusters and their exemplars through distance. The proposed approach of exemplars is conceptually similar to K-means’ cluster centroids. This work introduces an improved activation function, facilitating a smooth transition from soft-max to max functions. Experimental results, assessed via homogeneity and completeness metrics, demonstrate the algorithm’s effectiveness, especially with blob-shaped datasets, although stability issues related to local minima are noted. Comparisons with Gaussian mixture models, k-means models, and affinity propagation show AutoClustering’s performance.

As spike sorting is an unsupervised problem, all methods were trained and evaluated on the full set of spikes per dataset. No artificial train/test split was applied, as this would not reflect the intended use case in practice and would reduce the quality of the learned embeddings leading to lower performance. Moreover, deep clustering algorithms are based on autoencoders and do not require the labels during training; thus, there is no overfit phenomenon which would require a train/test split.

### Performance Metrics

A metric has to be chosen to assess the performance of these algorithms in analyses and one of the most commonly used metrics is accuracy. Although it has seen use in evaluating spike sorting techniques (Radmanesh et al., 2022; Eom et al., 2021), spike sorting is an inherently unsupervised task rendering accuracy unsuitable. Moreover, due to the different firing rates of neurons, spike sorting is inherently applied to imbalanced clusters and it has been thoroughly demonstrated that accuracy is not a suitable metric for such data (Weiss 2004; Wegier & Ksieniewicz 2020; Sun et al., 2009; Joshi et al., 2001).

Rather than relying on a single metric, we opted to use several metrics to capture the performance of these methods from a variety of angles helping us avoid evaluation bias. A method demonstrating high scores across all 6 performance metrics indicates a robust ability for clustering. This group of metrics allows us to evaluate the performance beyond the single concept of matching between true and predicted labels, through the use of internal metrics the separation and shape of the predicted clusters can be assessed (Arbelaitz et al., 2013).

Six metrics were employed for the evaluation of performance: Adjusted Rand Index (ARI), Adjusted Mutual Information (AMI), V-Measure (VM), Calinski-Harabasz Score (CHS), Davies-Bouldin Score (DBS), and Silhouette Score (SS). Each of these methods is shortly described in Table 1 along with an interpretation of its internal workings, range and type. As clustering follows feature extraction in spike sorting, internal metrics (Rendón et al., 2011) also reflect the separability imparted by the feature extraction algorithm as they evaluate the compactness and separation of the clusters in a given space without requiring ground truth labels. Conversely, external metrics evaluate the correspondence between the true labels and the predicted labels.

**Table 1:** A short description of each performance evaluation metric, specifying its type and range

Name	Type	Description	Range [worst, best]
ARI	External	Agreement between true and predicted labels using mutual information using a pairwise comparison, with an added normalization to account for random assignments.	[−1, 1]
AMI	External	Agreement between true and predicted labels using mutual information, with an added normalization to account for random assignments.	[0, 1]
Purity	External	Percentage of data points assigned to the correct class, assuming each cluster is labeled by majority vote, obtains the proportion of correctly assigned points.	[0, 1]
DBS	Internal	Ratio of within-cluster scatter to between-cluster separation, assessing cluster compactness and separation.	(Inf, 0]
CHS	Internal	The fraction between the dispersion between clusters and the dispersion within clusters, evaluating how distinct and compact the clusters are.	[0, Inf)
SS	Internal	Average of individual scores per data point that compare intra-cluster closeness with nearest-cluster separation, indicating overall clustering quality.	[−1, 1]

As deep clustering algorithms have both the abilities of feature extraction and clustering algorithms. Their comparison with more traditional pipelines from the perspective of these metrics allows for multiple options. External metrics require both the true labels (which may be provided by synthetic datasets) and the predicted labels (obtained by the clustering algorithm). Conversely, internal metrics require the input space and the predicted labels from clustering. As such, for traditional spike sorting, a clustering algorithm has to be applied after the feature extraction such as K-Means (MacQueen 1967) and DBSCAN (Ester et al., 1996) to obtain the predicted labels, whereas deep clustering does not require a step of feature extraction.

External metrics have the inherent disadvantage of requiring true labels which limit their usability. While internal metrics do not require true labels, they make assumptions about the cluster shape. These internal metrics provide higher scores for dense and well-separated clusters through their computations based on intra-cluster and inter-cluster distances. This implies that correct clustering labels might receive lower scores if the input data does not respect these assumptions.

#### External Metrics

The Rand Index (RI) (Fowlkes & Mallows 1983) is an external metric that makes pairwise comparisons between the predicted and true labels to find the amount of agreement between them. It considers two cases whether a pair of labels is in agreement, both data points are in the same/different clusters, and they disagree. ARI (Vinh et al., 2010; Steinley 2004; Hubert & Arabie 1985; undefined) is the extended version of RI with an improvement that allows it to account for random labellings. These metrics are computed through the following formulas:

1 $$\:\begin{array}{c}RI=\:\frac{agreements}{agreements+disagreements}\end{array}$$

2 $$\:\begin{array}{c}ARI=\:\frac{RI-ExpectedRI}{MaxRI-ExpectedRI}\end{array}$$

Here, *ExpectedRI* is the expected score if clusters were assigned randomly, estimated via a contingency table using permutations, *MaxRI*is 1, the maximum value of the score (Hubert & Arabie 1985).

Mutual Information (MI) measures the dependence between two clusters. AMI is an extended version of MI that with an improvement that allows it to account for random labellings and additionally it also contains the normalization (Vinh et al., 2010 undefined ;Vinh et al., 2009; Lazarenko & Bonald 2021) of Normalized Mutual Information. These metrics are computed through the following formulas:

3 $$\:\begin{array}{c}MI\left(U,\:V\right)={\sum\:}_{i=0}^{\left|U\right|}{\sum\:}_{j=0}^{\left|V\right|}\frac{\left|Ui\cap\:Vj\right|}Nloglog\:\frac{N\left|Ui\cap\:Vj\right|}{\left|Ui\right|\left|Vj\right|}\end{array}$$

4 $$\:\begin{array}{c}AMI=\:\frac{MI\left(U,\:V\right)-E\left(MI\left(U,V\right)\right)}{average\left(H\left(U\right),H\left(V\right)\right)-E\left(MI\left(U,V\right)\right)}\end{array}$$

Here, *U* and *V* are the two clusters, *N* is the total number of data points, *|X|* is the size of a given subset *X*, and *H* is the entropy.

Purity (Rendón et al., 2011; Manning et al., 2008) is computed as the division between the sum of the maximum intersections between the true and predicted labels for each cluster and the total number of samples, essentially it outputs the percentage of samples clustered correctly as the measure of many data points of the predicted labels belong to a single true cluster. Through its definition, Purity has the disadvantage of not penalizing overclustering. A clustering in which each data point is assigned to a different cluster, essentially having as many clusters as data points, receives a perfect score. This implies that the fewer data points there are in clusters, the higher the score which means that imbalanced datasets will receive a higher score due to the smaller clusters. This metric is computed through the following formula:

5 $$\:\begin{array}{c}Purity=\frac1N{\sum\:}_{i=1}^kmax\left|C_i\cap\:L\right|\end{array}$$

Here, *N* represents the total number of samples in the dataset, *k* is the number of clusters in the set of predicted labels, *C*_*i*_ represents the samples of a cluster, *i*, of the predicted set of labels and *L* is the set of true labels.

#### Internal Metrics

DBS (Halkidi et al., 2001; Caliński 1974; Davies & Bouldin 1979) utilizes the size of clusters (as the mean distance among all data points of said clusters) and the distance between clusters. Through the division of these two terms, a similarity measure is obtained. DBS is computed as the average similarity of all clusters. The main challenge of DBS is that the score range is reversed, meaning that lower score values indicate a higher performance and additionally it has no upper bound. This metric is computed through the following formulas:

6 $$\:\begin{array}{c}R_{i,j}=\frac{s_i-s_j}{d_{i,j}}\end{array}$$

7 $$\:\begin{array}{c}DBS=\:\frac1k{\sum\:}_{i=1}^kmaxR_{iJ}\end{array}$$

Here, *R* represents the similarity between clusters *i* and *j*, *s*_*i*_ is the mean of all distances between the points of cluster *i* and its centroid, *d*_*i, j*_ is the distance between clusters *i* and *j* given by their centroids, and *max(R*_*i, j*_*)* is the maximum similarity of clusters *i* and *j*.

CHS (Rendón et al. 2011; Rosenberg & Hirschberg 2007) is computed as the division between intra-cluster and inter-cluster dispersions; the dispersion computation is based on the sum of squared distances. The main challenge of CHS is that it has no upper bound, meaning that there is no indication of when a perfect clustering is obtained. This metric is computed through the following formula:

8 $$\:\begin{array}{c}CHS=\frac{tr\left(Bk\right)}{tr\left(Wk\right)}\ast\frac{n-k}{k-1}\end{array}$$

Here, *tr(X)* is the trace of the dispersion matrix (either between *Bk* or within *Wk*), *n* is the dataset size and *k* is the number of clusters.

SS (Rosenberg & Hirschberg 2007; Rousseeuw & Silhouettes 1987) for a single data point is computed as the average distance between that point and the rest of the data points of the cluster it belongs to and the average distance between that point and all the points of the closest different cluster. To obtain the SS of an entire dataset, the mean of all data points is computed. This metric is computed through the following formula:

9 $$\:\begin{array}{c}SS=\:\frac{b-a}{max\left(a,b\right)}\end{array}$$

Here, *b* is the average of all distances between a point in cluster *i* and all points of the closest cluster *j*, and *a* is the average of all distances between a point in cluster *i* and all other points in the same cluster.

### Synthetic Data

The 95 synthetic datasets, also called simulations, used in this study were created based on “in vivo” recordings from a monkey brain by the Department of Engineering, University of Leicester, UK, and are publicly available. We have chosen to analyze the proposed methods on these 95 datasets (Pedreira et al., 2012) from the perspective of 6 performance metrics. We did not participate in their design or parameter selection. Consequently, the properties of the datasets were predefined and could not be altered within the scope of our benchmarking.

From these recordings, 594 unique spike shapes (Pedreira et al., 2012) were extracted that were used in the generation of these synthetic datasets. The initial spikes obtained were sampled at a sampling frequency of 96 kHz resulting in spikes of 316 samples, which were then downsampled to 24 kHz resulting in 79 samples. The datasets have been generated in such a way that no spikes can overlap having at least 0.3ms between them. Each of these datasets provides ground labels which allow for the evaluation of performance using external metrics as well as internal. The datasets were created with varying cluster counts, each having a unique count from 2 to 20 clusters. Thus, there are 5 different datasets for each cluster count in this range. To increase the complexity of these datasets, each contains a single multi-unit cluster, while the rest are single-unit clusters.

Each multi-unit cluster consists of 20 different spike shapes from 20 different neurons at about 50–140 μm away from the electrode each with a mean firing rate of 0.25 Hz following a Poisson distribution (with a total firing rate of 5 Hz). Due to the larger distance from the electrode, the amplitudes of the spikes from multi-unit cluster was fixed to 0.5. Conversely, single-unit clusters consist of a single unique spike shape from a neuron at about 0–50 μm away from the electrode with its mean firing rate in the 0.1–2 Hz following a Poisson distribution. The amplitudes of spikes of single-unit clusters has been scaled in the 0.9–2.9 range following a normal distribution.

**Fig. 1:**
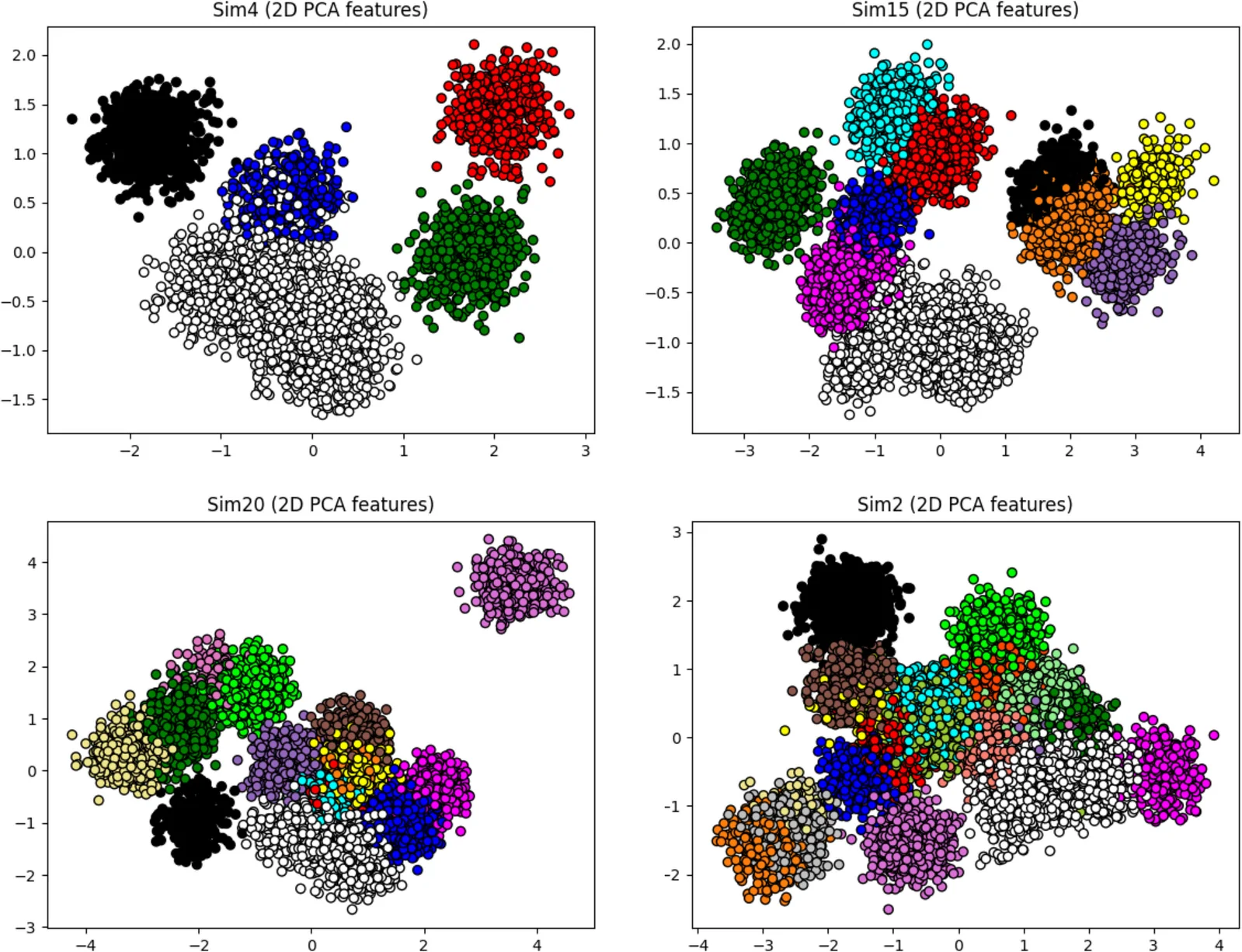
Synthetic datasets presented with PCA and ground truth labels. Four different simulations were reduced to a2-dimensional space using PCA. The colors represent the true clusters indicating that PCA is unable to find a set of features that offer cluster separability

The complexity of these datasets was confirmed by the fact that no clustering algorithm was able to identify more than 10 clusters out of the maximum of 20 that are available in these datasets (Pedreira et al., 2012). Out of the 95 datasets, 4 was chosen for an initial comparative analysis with increasing cluster counts (and number of samples) to evaluate the performance for different levels of complexity. In Fig. 1, each of these 4 datasets are shown in the 2-dimensional space obtained through applying PCA. These 4 datasets have the following characteristics:

- Simulation 4 (Sim4 – Fig. 1) contains 5127 spikes grouped in 4 single-unit clusters and a multi-unit cluster (in total 5 clusters).
- Simulation 15 (Sim15 – Fig. 1) contains 9683 spikes grouped in 9 single-unit clusters and a multi-unit cluster (in total 10 clusters).
- Simulation 20 (Sim20 – Fig. 1) contains 11,186 spikes grouped in 14 single-unit clusters and a multi-unit cluster (in total 15 clusters).
- Simulation 2 (Sim2 – Fig. 1) contains 12,784 spikes grouped in 19 single-unit clusters and a multi-unit cluster (in total 20 clusters).

#### Data Preprocessing

Besides the traditional scaling and shuffling of data, a first step of preprocessing that might improve spike sorting performance is the alignment of spikes (the necessity of this step is dependent upon the spike detection. The following expression was employed for to align all spikes to a given index:

10 $$\:\begin{array}{c}ne{w\_start}_{spike}=\:ol{d\_start}_{spike}-\left(index_{align}-peak_{spike}\right)\end{array}$$

**Fig. 2:**
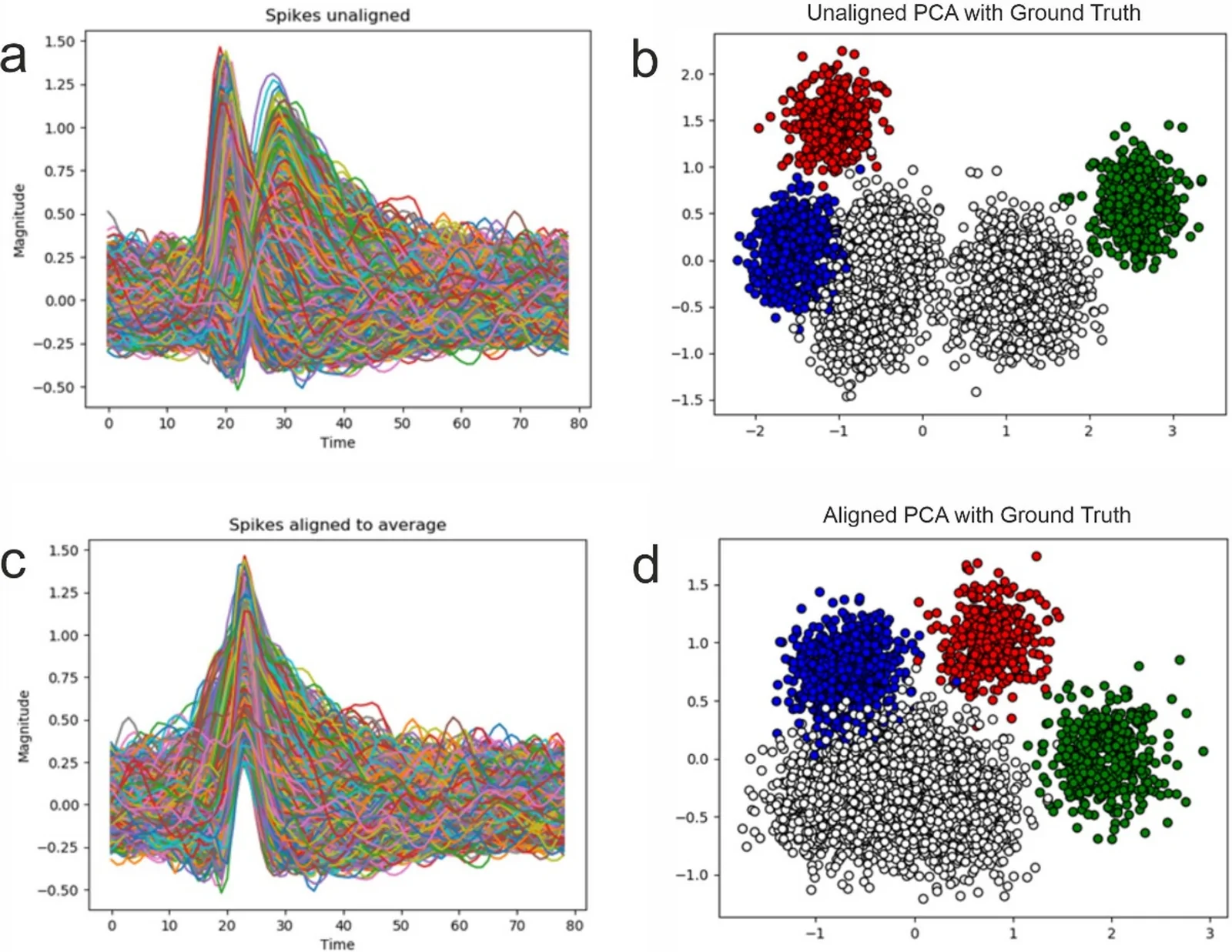
Impact of alignment. PCA applied on Sim29 with and without alignment. The white cluster is kept together but the overlap with the blue cluster remains

The starting index of a spike waveform in the recorded signal is given by the *old_start* term, which must be shifted to *new_start* to align all spikes to the same *index*. The reference point for alignment can be any index of the spike. However, the superior choice regarding performance is the maximum peak of the spike, also called the amplitude which is represented by the *peak*term. This formula offers flexibility as any reference point could be chosen for alignment (Dipalo et al. 2017). The impact of alignment can be viewed in Fig.2.

### Real Data

The spe‑1 dataset (Marques-Smith et al., 2020, 2018) provides a rare ground‑truth resource by recording from the same cortical neuron in rats anesthetized with urethane using simultaneous patch‑clamp and high‑density 384‑channel CMOS extracellular probes. Across primary motor and somatosensory cortex, 43 neurons were targeted out of which 38 were recorded in cell‑attached mode and 5 in whole‑cell, yielding clear extracellular action potentials for 21 neurons—10 of which exhibited peak‑to‑peak amplitudes over 50 µV—thereby enabling direct validation of spike‑sorting algorithms. For each neuron, the dataset includes high‑pass–filtered (300 Hz) extracellular voltage traces alongside intracellular patch‑clamp recordings.

Two datasets were chosen from the 43 available, specifically c28 and c37. The raw recordings were band-pass filtered in the 300–7000 Hz range, and the spikes were extracted using the traditional amplitude thresholding of the standard deviation of the filtered signal multiplied by a factor of 4. These datasets are simultaneous extracellular and intracellular recordings, providing ground-truth spike timings for one neuron (or one cluster). Yet, due to the extracellular nature of the recording, the total number of neurons (or clusters) in the recording is unknown.

## Results

Although spike sorting is an unsupervised task, external clustering metrics can be used when ground-truth labels are available. For the synthetic datasets, all ground truth labels are known, while for the real datasets (evaluated in a one-vs-rest manner), the spikes of a single neuron (or cluster) are known. In all other cases, only internal metrics are applicable, which assess cluster compactness and separation without labels but can vary across different feature spaces. For a comprehensive analysis, we report both external and internal metrics, allowing performance to be evaluated both in terms of label agreement and cluster quality, respectively.

### Performance Evaluation of Individual Synthetic Datasets

We begin the analysis with the evaluation of the four selected datasets (Pedreira et al., 2012). Each of the deep clustering methods was run on these datasets and we make a comparative analysis of these methods against traditional feature extraction algorithms combined with K-Means. All deep clustering approaches have been created with the same neural network architecture of [60,40,20] with an embedding size of 10 and have been trained on the spike data on batches of 256. As with K-Means, the true number of clusters has been provided in order to make a fair comparison among clustering abilities. Other parameters, such as the learning rate and specific parameters of the method are presented in Table2. These have been found through a grid search as the best performing across the datasets. A small subset of parameters tested through the grid search can be viewed in Table S1 in the Supplementary Material.

**Table 2:** Parametrization of deep clustering approaches

Algorithm	Pretrain learning rate	Clustering learning rate	Other parameters
ACeDeC	1e-3	1e-3	Initialisation: ‘acedec’
AEC	1e-5	1e-2	-
DCN	1e-3	-	-
DDC	1e-3	-	Ratio: 0.1
DEC	1e-3	1e-4	Alpha: 0.25
DeepECT	1e-2	1e-4	Max leaves: 20
DipDECK	1e-2	1e-3	Merge threshold: 0.9
DipEncoder	1e-2	1e-4	-
DKM	1e-3	1e-5	-
IDEC	1e-3	1e-4	Alpha: 0.25
N2D	1e-2	-	Manifold: t-SNE
VaDE	1e-2	1e-3	-

The results obtained on the simpler case of Sim4, which contains only 5 clusters, are presented in Table 3. For this dataset, DipDECK and DCN achieved the best performance across the metrics. Traditional methods performed moderately well outperforming the other deep clustering methods with DeepECT and AEC having significantly lower performances.

**Table 3:** Comparison of feature extraction methods on Sim4 (containing 5 clusters) from the perspective of the six performance evaluation metrics

Algorithm	ARI	AMI	Purity	SS	CHS	DBS
PCA	0.573	0.796	0.913	0.257	**2439.37**	1.413
ICA	0.613	0.8	**0.914**	0.254	2403.811	1.437
Isomap	0.557	0.791	0.913	0.252	2417.936	1.421
LLE	0.601	0.747	0.828	0.012	742.938	1.117
t-SNE	0.566	0.771	0.895	0.182	1680.762	1.686
DM	0.597	0.807	**0.914**	0.257	2425.074	1.425
ACeDeC	0.634	0.77	0.905	0.208	2083.272	1.766
AEC	0.358	0.61	0.818	0.173	1935.731	2.278
DCN	0.77	**0.859**	0.913	0.305	1997.801	1.046
DDC	0.386	0.651	0.904	0.175	1501.293	2.29
DEC	0.567	0.792	**0.914**	0.213	2174.633	1.689
DKM	0.526	0.734	0.903	0.234	2291.403	1.486
DeepECT	0.134	0.439	0.879	0.006	492.392	4.021
DipDECK	**0.791**	0.852	0.903	**0.331**	2429.059	**1.029**
DipEncoder	0.523	0.763	0.911	0.201	2100.436	1.806
IDEC	0.563	0.786	**0.914**	0.211	2158.338	1.715
N2D	0.471	0.696	0.879	0.208	1955.052	1.622
VaDE	0.494	0.709	0.826	0.204	1844.804	1.547

As the number of clusters increases, the complexity does so as well, which favours the deep clustering methods. DDC emerges as the top performer for this dataset with the high scores across all metrics, followed closely by DEC and IDEC. Slightly lower performance than these methods, yet still higher than traditional methods were obtained by AceDeC, DCN, DipEncoder and VaDE. At the same time, a subset of deep clustering methods were on par with the traditional methods, specifically DKM, DipDECK and N2D. As with the case of Sim4, AEC and DeepECT have the lowest scores for this dataset as well.

In Table 5, for Sim20, which contains 15 clusters, IDEC obtained the highest scores, followed closely by DEC, DCN and DM. When comparing the scores obtained on Sim15 (Table 4) and Sim20 (Table 5), traditional methods had a severe degradation in performance as the number of clusters increased. The high performance of these deep clustering methods demonstrate their advantage on more complex datasets. However, there are deep clustering methods that had similar or lower performance when compared with the traditional methods. N2D and DKM had a higher performance than PCA, ICA or LLE, yet comparable to that of Isomap and t-SNE, while AEC and DeepECT continue to underperform. DipEncoder and Vade had slightly higher performances than that of Isomap, yet still lower than the top performing deep clustering methods or DM.

**Table 4:** Comparison of feature extraction methods on Sim15 (containing 10 clusters) from the perspective of the six performance evaluation metrics

Algorithm	ARI	AMI	Purity	SS	CHS	DBS
PCA	0.717	0.852	0.895	0.246	3178.53	1.515
ICA	0.706	0.84	0.888	0.235	2997.72	1.563
Isomap	0.8	0.925	0.97	0.298	4042.44	1.309
LLE	0.676	0.894	0.835	0.262	2479.79	**1.043**
t-SNE	0.807	0.94	**0.976**	0.297	**4069.64**	1.302
DM	0.909	0.946	0.893	0.227	1114.37	1.844
ACeDeC	0.799	0.932	0.974	0.287	3987.26	1.365
AEC	0.317	0.545	0.592	0.061	1632.92	3.434
DCN	0.879	0.942	0.953	0.297	3479.01	2.51
DDC	**0.949**	**0.959**	0.95	**0.34**	4057.98	1.123
DEC	0.93	0.961	0.953	0.33	3782.34	1.122
DKM	0.724	0.888	0.927	0.275	3564.71	1.446
DeepECT	0.442	0.695	0.845	0.056	1457	3.623
DipDECK	0.67	0.833	0.826	0.22	2749.83	2.09
DipEncoder	0.873	0.899	0.924	0.27	3350.92	2.343
IDEC	0.93	0.961	0.953	0.33	3780.37	1.12
N2D	0.762	0.908	0.949	0.27	3688.3	1.63
VaDE	0.859	0.923	0.932	0.32	3796.82	1.284

**Table 5:** Comparison of feature extraction methods on Sim20 (containing 15 clusters) from the perspective of the six performance evaluation metrics

Algorithm	ARI	AMI	Purity	SS	CHS	DBS
PCA	0.491	0.738	0.806	0.157	3365.14	2.226
ICA	0.535	0.75	0.831	0.169	3418.442	2.32
Isomap	0.628	0.818	0.889	0.24	4217.91	2.397
LLE	0.327	0.631	0.608	0.112	1602.482	2.95
t-SNE	0.687	0.898	**0.948**	0.3	4891.882	1.654
DM	0.776	0.886	0.807	0.189	896.797	2.051
ACeDeC	0.713	0.863	0.911	0.205	4421.748	2.397
AEC	0.255	0.499	0.555	0.05	1714.12	6.292
DCN	0.637	0.821	0.844	0.181	3689.184	4.013
DDC	0.833	0.893	0.844	**0.367**	**5863.07**	**1.023**
DEC	0.811	0.875	0.8	0.185	3401.911	3.176
DKM	0.595	0.808	0.859	0.202	4162.171	2.386
DeepECT	0.414	0.612	0.678	0.102	1649.436	2.985
DipDECK	0.814	0.835	0.862	0.207	4134.466	2.637
DipEncoder	0.714	0.808	0.805	0.153	3095.602	2.973
IDEC	**0.874**	**0.916**	0.877	0.23	3821.42	3.094
N2D	0.648	0.836	0.897	0.25	4257.642	2.482
VaDE	0.756	0.887	0.932	0.287	4445.648	1.709

In the most complex dataset, VaDE demonstrates its ability to cluster obtaining remarkably high scores outperforming all other methods. DEC and IDEC maintained a high performance, scoring slightly lower than VaDE. DDC had a high performance as well but was lower than VaDE, DEC and IDEC and comparable to that of DM, while still outperforming all other methods (Table 6). The other deep clustering algorithms had scores close to or lower than those of traditional methods. The lowest scores were obtained by AEC and DeepECT.

**Table 6:** Comparison of feature extraction methods on Sim2 (containing 20 clusters) from the perspective of the six performance evaluation metrics

Algorithm	ARI	AMI	Purity	SS	CHS	DBS
PCA	0.438	0.715	0.766	0.086	1937.573	2.765
ICA	0.446	0.71	0.753	0.081	1872.313	3.033
Isomap	0.607	0.81	0.865	0.17	2452.905	2.359
LLE	0.672	0.84	0.703	0.143	1699.404	**1.133**
t-SNE	0.62	0.868	**0.922**	0.183	2806.787	2.263
DM	0.737	0.879	0.79	0.18	1800.642	1.627
ACeDeC	0.586	0.806	0.878	0.19	2811.504	2.383
AEC	0.196	0.445	0.488	−0.027	757.366	9.655
DCN	0.578	0.797	0.723	0.101	1874.234	3.401
DDC	0.739	0.845	0.749	0.28	**3809.325**	1.298
DEC	0.855	0.879	0.837	0.189	2410.605	3.402
DKM	0.492	0.727	0.75	0.13	2167.347	2.952
DeepECT	0.414	0.665	0.723	0.072	2081.063	3.103
DipDECK	0.507	0.751	0.779	0.117	2268.812	2.788
DipEncoder	0.429	0.704	0.714	0.069	1877.737	2.872
IDEC	0.86	0.882	0.84	**0.207**	2422.558	3.051
N2D	0.485	0.763	0.797	0.145	2387.816	2.672
VaDE	**0.919**	**0.91**	0.884	0.183	1982.449	2.2

Our analysis on individual datasets shows that there are a subset of deep clustering algorithms that are suitable for the task of spike sorting, specifically DDC, DEC, IDEC and VaDE which obtained high scores across these datasets. DDC expressed its highest scores on the datasets with low to medium number of clusters, while the DEC, IDEC and VaDE were top performers for datasets with a medium to high number of clusters.

The ability of deep clustering methods to separate the multi-unit cluster can be viewed in Fig S1 in the Supplementary Material. In this figure, the multi-unit cluster has the same localization in all plots as the same 2D PCA projected feature space is shown and can be identified as the red cluster in the plot labelled as ‘Ground truth’. Through comparison with Table 5, it is clearly visible that the methods which obtained the highest scores are the ones that were also capable of correctly identifying the multi-unit cluster, specifically DCN, DDC, IDEC and VaDE. Moreover, these methods also generated the least overlap between clusters (AEC and N2D generated highly overlapping clusters due to overclustering). Even though these methods have obtained the highest scores, they are not perfect, and this can be attributed to underclustering as some ground truth clusters are merged into a single predicted cluster.

Additional analyses have been included in the Supplementary Material. In Table S3, the performance of all algorithms is shown on the Sim2 dataset in a one-vs-rest label setup with one true single-unit kept while all the rest have been attributed the same label. In Table S4, the impact of the choice of the number of clusters parameter on the performance of the IDEC algorithm on the Sim2 dataset is shown. As expected, when the correct number of clusters is given the performance is highest, while more distal values obtain lower performances. However, the question of cluster number identification is a separate methodological challenge that has been extensively addressed in the clustering literature through techniques such as the Elbow method, Gap statistic, silhouette analysis, and information criteria approaches.

### Performance Evaluation of all Synthetic Datasets

A thorough analysis of performance requires varying levels of complexity in the data used. Our analysis based on 95 datasets (Pedreira et al. 2012) containing a range of cluster counts and spike shapes allows for an extensive evaluation of performance.

**Fig. 3:**
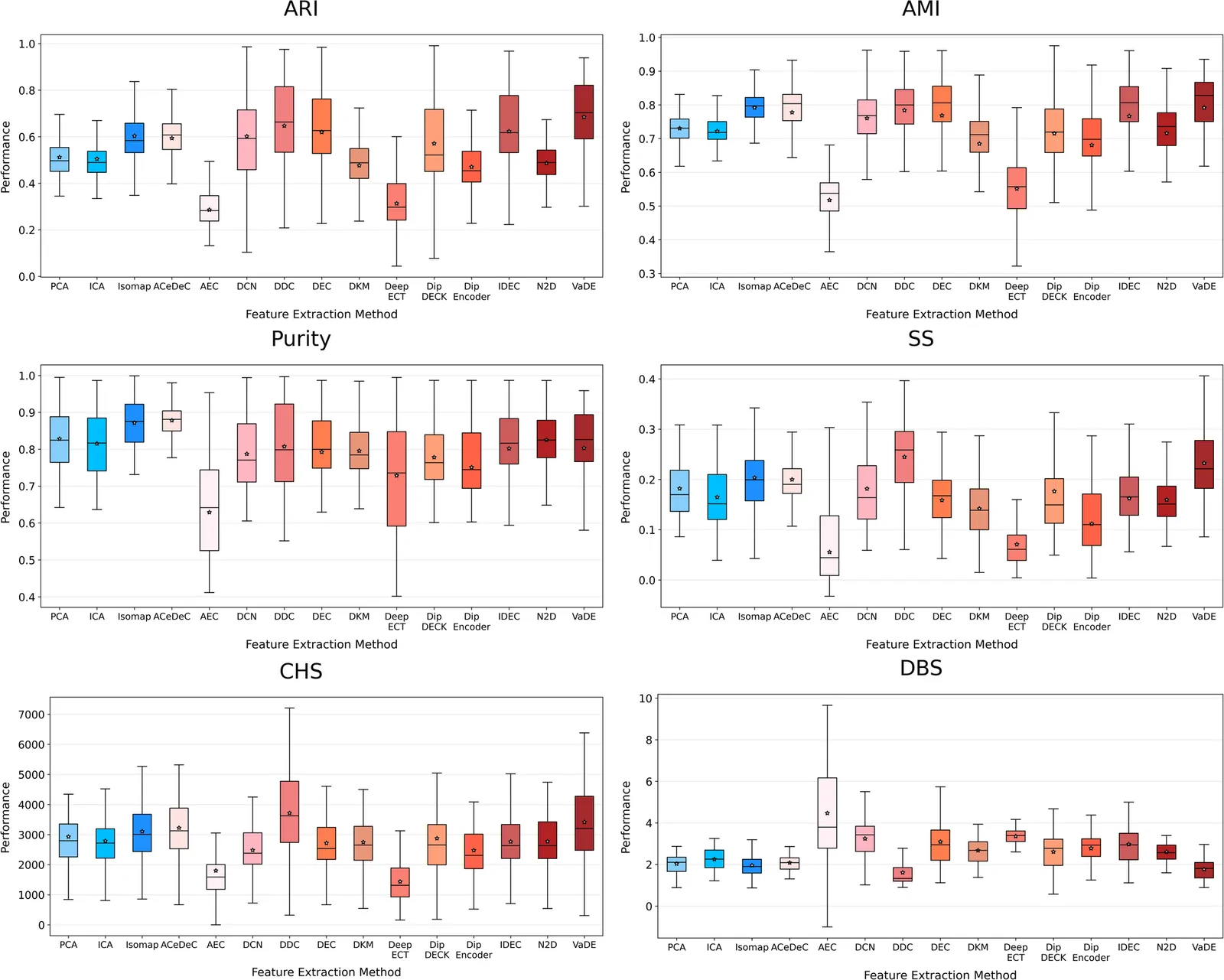
Performance evaluation of all feature extraction methods for all 95 datasets (the star represents the mean value, while the middle line represents the median value)

The results obtained by each method across all 95 datasets by each performance metric are shown in Fig. 3 and complemented through a Borda aggregation-based ranking. Through visual inspection of the performance distribution, it is observable that ACeDeC, DDC and VaDE are the methods that have higher mean and median scores while also expressing a narrow distribution that show better performance than the traditional methods. However, there are methods that do not manage to reach the performance of these simpler methods, specifically AEC and DeepECT consistently obtain poor scores.

The Borda aggregation-based ranking of the methods according to their performance on each metric is presented in Table 7. Indicating that indeed a subset of deep clustering algorithms are suitable approches to spike sorting, specifically VaDE, DDC and AceDeC. However, AEC, DeepECT and DipEncoder are not as they obtain lower performances than the most simple and traditional approach of combining PCA and K-Means. There are deep clustering methods that manage to outperform linear feature extraction methods in combination with K-Means, however when compared to Isomap, a non-linear manifold approach, they fall behind. These methods are DCN, DEC and IDEC.

**Table 7:** Borda ranking by each performance metric across all 95 datasets

Method	ARI	AMI	Purity	SS	CHS	DBS
1	VaDE	t-SNE	t-SNE	DDC	DDC	DM
2	t-SNE	VaDE	Isomap	VaDE	t-SNE	DDC
3	DDC	DM	ACeDeC	t-SNE	ACeDeC	t-SNE
4	IDEC	DEC	PCA	DM	Isomap	VaDE
5	DEC	Isomap	IDEC	Isomap	VaDE	Isomap
6	DM	IDEC	N2D	ACeDeC	PCA	PCA
7	Isomap	DDC	DEC	PCA	ICA	ACeDeC
8	ACeDeC	ACeDeC	VaDE	DCN	DipDECK	ICA
9	DCN	DCN	ICA	IDEC	IDEC	LLE
10	DipDECK	PCA	DDC	DEC	N2D	DipDECK
11	LLE	LLE	DKM	DipDECK	DKM	N2D
12	PCA	DipDECK	DM	ICA	DEC	DKM
13	ICA	ICA	DCN	N2D	DipEncoder	IDEC
14	DKM	N2D	LLE	LLE	DCN	DipEncoder
15	DipEncoder	DipEncoder	DipDECK	DKM	LLE	DEC

**Fig. 4:**
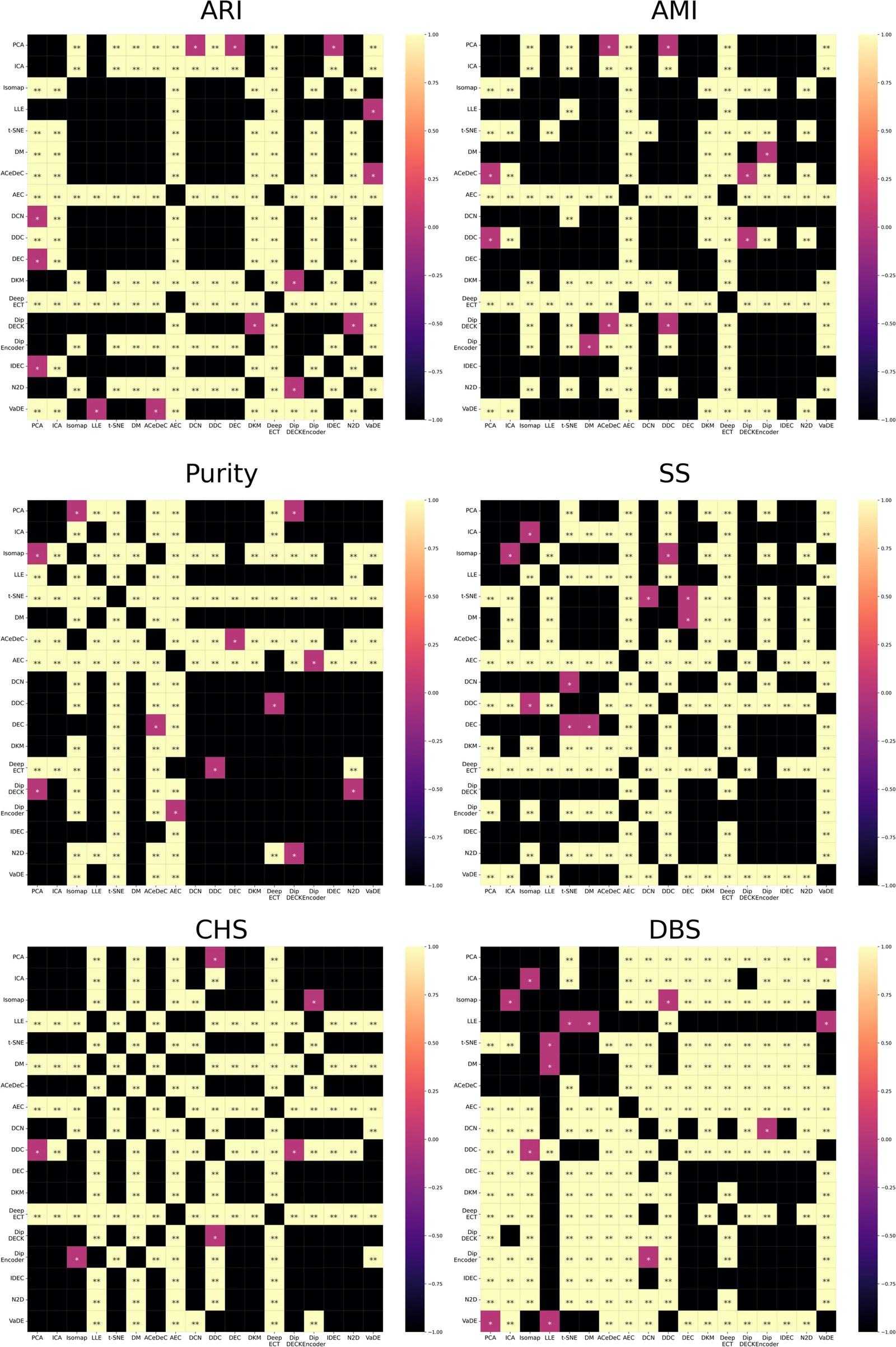
P value of t-tests (with a Bonferroni correction for all pairwise comparisons and all metrics) for each of the metric on all 95 simulations (** represents *p* < 0.01, * represents 0.01 < *p* < 0.05, while no text represents 0.05 < *p*)

In Fig. 4, a statistical analysis can be reviewed carried out via Bonferroni corrected (for all pairwise comparisons and all metrics) t-tests. This validates our results further by indicating which methods shown a statistically significant difference. The three best performing methods, ACeDeC, DCN and VaDE do not have a significant difference among themselves while having a significant difference to all other methods except Isomap, when considering the ARI, AMI and SS metrics. The Purity metric indicates only that Isomap, ACeDeC and AEC are statistically different to all other methods.

As expected, PCA and ICA do not show a statistical difference. The CHS metric (which has no upper bound) clearly shows that the methods with the lowest performances, AEC and DeepECT, are statistically different from all other methods due to their significantly lower scores. However, the DBS metric (which has a reversed range) shows again that VaDE and DCN, the better performing algorithms do not have a statistical difference.

### Performance Evaluation on Real Datasets

The real datasets used in this analysis contain ground truth labels for the spikes of a single neuron, translating into a single cluster. This has been achieved through a simultaneous recording of intracellular and extracellular activity. As such, the performance of deep clustering algorithms can be evaluated using external metrics as well for this ground truth, while internal metrics evaluate the compactness of the identified clusters in the original spike space. External metrics (ARI, AMI, Purity) were computed in a one-vs-rest manner, treating all unlabeled spikes as a single “other” class. This evaluation underestimates the absolute performance of algorithms that may correctly separate multiple neurons but where only one cluster is validated. To address this limitation, we also report internal clustering metrics, which require no ground truth and instead evaluate cluster compactness and separation. Combining both types of metrics provides a more comprehensive understanding of algorithm performance in datasets with partial labeling.

The scores obtained by each deep clustering algorithm and the traditional feature extraction and clustering approaches are presented in Tables 8 and 9 for the c28 and c37 real datasets, respectively. In these tables, the highest scores have been highlighted through bolding. Our evaluation indicates that a subset of deep clustering approaches provide a high performance for real datasets as well, specifically DDC and VaDE. However, when considering the results obtained on synthetic datasets, ACeDeC, DEC and IDEC do not perform as well on real datasets, while DDC and DipDECK emerge as highly performant for real datasets when their performance on synthetic datasets was lacking. On two out of the six metrics for the c28 real dataset, the highest performance was obtained by the Isomap and K-Means combination; nevertheless, the performance on c37 dataset is considerably lower indicating that this high performance may be an edge case. Although, t-SNE and DM obtained high scores for the synthetic datasets, their performance was significantly lower in handling real datasets, lower than PCA.

**Table 8:** Performance analysis of deep clustering methods on the c28 real dataset. Bold values represent the highest score

Algorithm	ARI	AMI	Purity	SS	CHS	DBS	Time(s)
PCA	0.228	0.291	0.922	0.134	3038.412	2.162	0.002 0.002
ICA	0.228	0.29	0.928	0.125	2928.826	2.28	0.02 0.001
Isomap	**0.631**	0.448	**0.943**	0.396	2751.06	1.921	15.830 0.001
LLE	0.134	0.248	0.906	0.37	2489.889	1.019	39.328 0.001
t-SNE	0.11	0.245	0.9	0.326	3367.399	**0.867**	13.70 0.001
DM	0.225	0.068	0.848	0.508	4.191	23.007	13.142 0.001
ACeDeC	0.225	0.288	0.924	0.089	2749.917	2.735	83.221
AEC	0.21	0.26	0.914	0.079	2627.648	2.948	211.378 0.430
DCN	0.583	0.387	0.924	0.329	2422.237	3.153	56.670 0.211
DDC	0.604	**0.471**	0.907	**0.461**	**6168.923**	1.085	26.940
DEC	0.235	0.307	0.935	0.116	2957.921	2.296	69.330 0.214
DKM	0.47	0.316	0.868	0.389	1545.155	1.325	147.397 0.193
DeepECT	0.233	0.27	0.928	0.046	1503.317	3.211	222.040 0.122
DipDECK	0.614	0.416	0.907	0.349	3538.857	2.031	63.764 0.186
DipEncoder	0.278	0.3	0.919	0.116	2830.488	2.428	158.385
IDEC	0.236	0.308	0.936	0.116	2958.062	2.295	123.659 0.197
N2D	0.117	0.25	0.907	0.092	2535.787	2.318	23.087
VaDE	0.604	0.432	0.926	0.392	3191.315	2.332	138.635 0.264

**Table 9:** Performance analysis of deep clustering methods on the c37 real dataset. Bold values represent the highest score of each metric

Algorithm	ARI	AMI	Purity	SS	CHS	DBS	Time(s)
PCA	0.326	0.388	0.954	0.171	1498.531	1.934	0.001 0.001
ICA	0.257	0.343	0.948	0.132	1341.377	2.238	0.01 0.001
Isomap	0.372	0.406	**0.957**	0.18	1486.68	1.864	2.826 0.001
LLE	0.26	0.353	0.948	**0.479**	1767.493	0.907	4.525 0.001
t-SNE	0.199	0.298	0.901	0.398	2184.416	**0.687**	4.457 0.001
DM	0.059	0.015	0.852	0.5	0.945	15.929	5.154 0.001
ACeDeC	0.314	0.386	0.954	0.161	1482.999	1.986	47.308
AEC	0.323	0.384	0.954	0.136	1278.648	2.38	88.012 0.181
DCN	**0.767**	**0.578**	0.955	0.309	1060.041	2.363	24.861 0.086
DDC	0.054	0.197	**0.957**	0.004	307.165	2.807	11.210
DEC	0.277	0.358	0.945	0.138	1368.288	2.257	31.986 0.106
DKM	0.509	0.369	0.878	0.324	897.834	1.604	32.539 0.097
DeepECT	0.259	0.282	0.907	0.058	617.227	3.258	97.596 0.053
DipDECK	0.713	0.55	0.939	0.431	**2205.745**	1.041	31.225 0.088
DipEncoder	0.189	0.272	0.864	0.128	1185.59	2.2	207.252
IDEC	0.28	0.36	0.946	0.139	1363.786	2.252	252.984 0.187
N2D	0.13	0.243	0.852	0.077	976.677	2.77	10.704
VaDE	0.653	0.504	0.934	0.384	1140.658	3.62	59.412 0.127

**Fig. 5:**
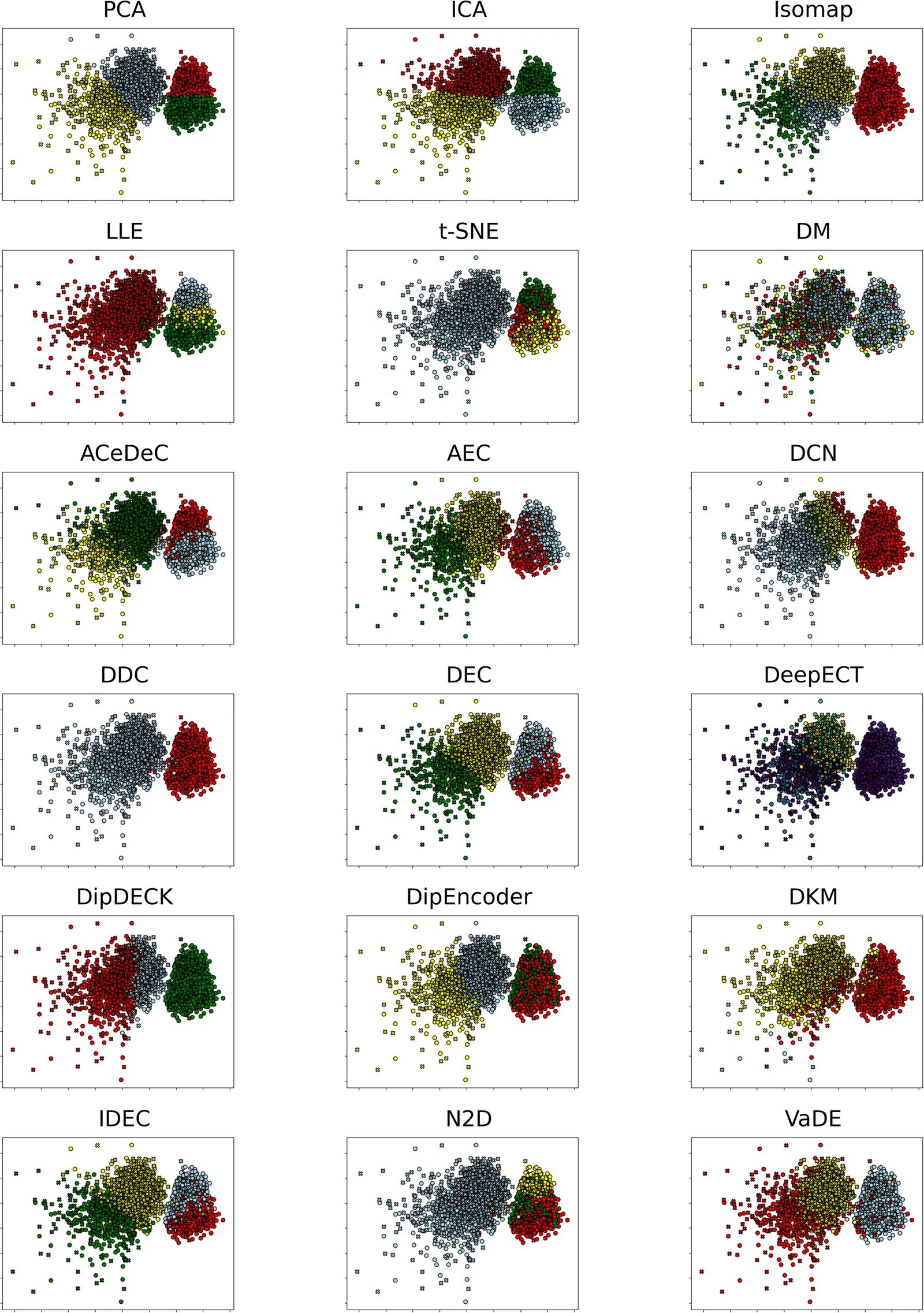
Deep clustering methods on the c28 real dataset. Colors represent the clustering labels and the ‘X’ markers represents the intracellular action potentials (also the ground truth) such that the amount of separability offered is easily observable (as many ‘X’ marked points as possible should have the same color)

**Fig. 6:**
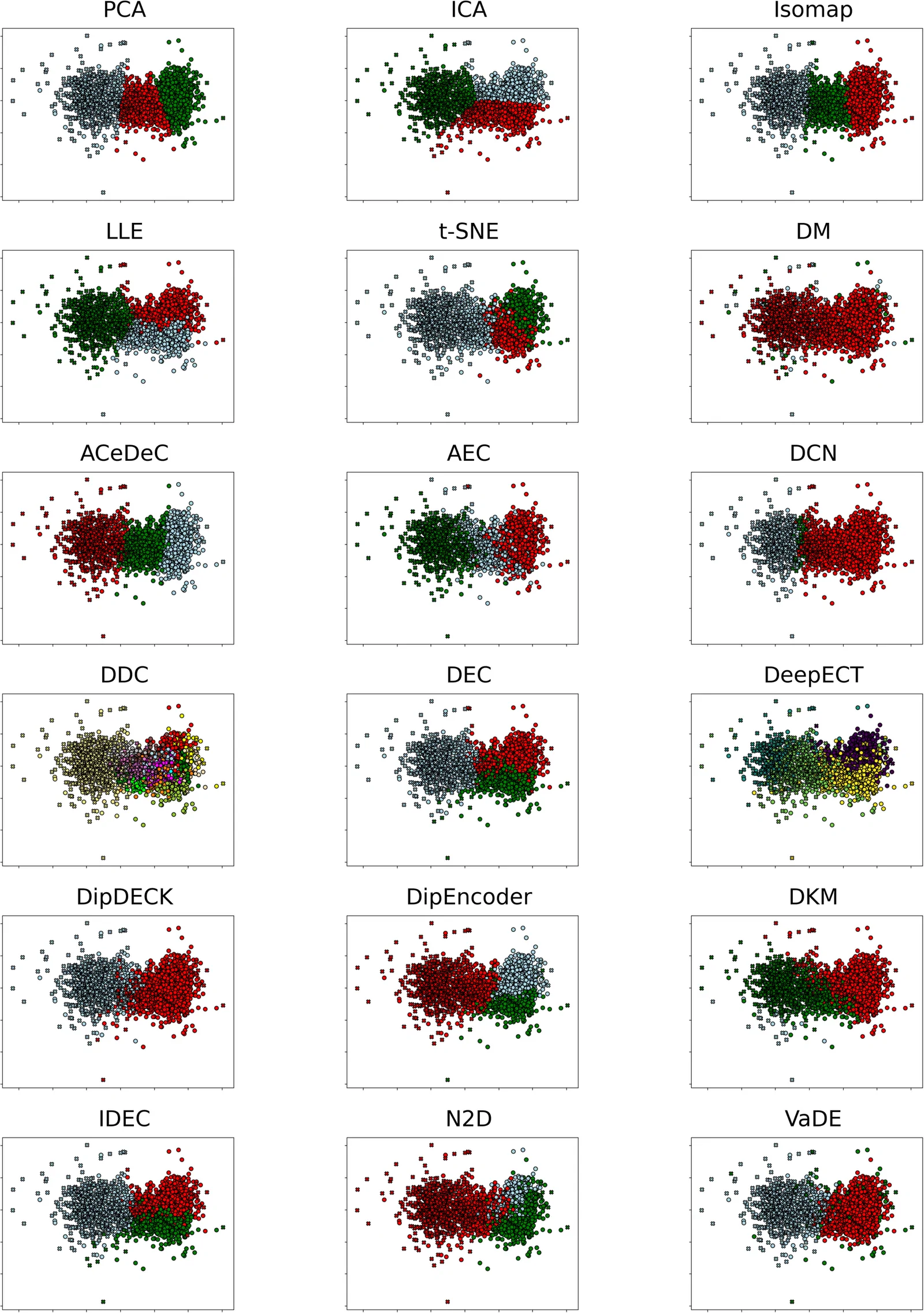
Deep clustering methods on the 37 real dataset. Colors represent the clustering labels and the ‘X’ markers represents the intracellular action potentials (also the ground truth) such that the amount of separability offered is easily observable (as many ‘X’ marked points as possible should have the same color)

A visual inspection of the clusterings obtained by these methods can be made through Figs. 5 and 6 for the c28 and c37 real datasets, respectively. Due to the embedding size of deep clustering algorithms (chosen for its high performance), direct visualization is unattainable. As such, using the same PCA 2-dimensional space to visualize the data, we provide here a visualization of clustering labels (through colors) obtained for each of the methods used in this analysis. The ground truth intracellular labels can also be viewed in this figure as they have been marked with ‘X’, while the rest of the spikes come from an arbitrary number of neurons have been marked with ‘O’.

A time analysis was included for both real datasets showing two values. For the traditional feature extraction methods, the first value is the running time of the feature extraction method, while the second value is the running time of K-Means. While for the deep clustering methods, where applicable, the first value is the training time and the second is the inference time, otherwise there is a single value for the training and inference times. The time values are shown as an average of 5 runs on an Apple M4 Pro. Although, the deep clustering methods require a longer training time, the inference time is under 1s. DCN and VaDE have shown a high performance on both synthetic and real datasets, however DCN has a significantly lower execution time. All deep clustering methods require a higher computational cost than traditional feature extraction methods.

A more detailed analysis of the results obtained by the best performing algorithm DCN on the c37 dataset can be found in the Supplementary Material. The true and predicted spikes are shown in Figure S2, while additional statistics about the result obtained can be viewed in Figure S3.

## Conclusions

Our study presents a comprehensive evaluation of deep clustering algorithms for spike sorting, comparing their performance against traditional feature extraction methods combined with K-Means from the perspective of six performance metrics on 95 synthetic datasets and 2 real datasets. Our analysis of performance on synthetic datasets demonstrates that certain deep clustering approaches, particularly ACeDeC, DDC, DEC, IDEC and VaDE, provide significant improvements over traditional methods, especially when the number of clusters and dataset complexity increases. While the real dataset performance indicates that out of these methods DDC and VaDE can offer a high performance regardless of data.

VaDE’s strong performance across datasets highlights the effectiveness of generative approaches for spike sorting, suggesting that modeling the underlying distribution of spike data contributes significantly to clustering and thus, neuron identification. DDC’s simpler density-based approach demonstrates the importance of accounting for varying cluster densities and shapes in neuronal recordings. The dual optimization of DEC and IDEC by combining the reconstruction loss with a clustering-specific loss in the autoencoder highlights their ability to simultaneously learn effective data representations and perform clustering when handling synthetic datasets, however their performance remains lacking when tackling real datasets. Our analysis also revealed that not all deep clustering methods are equally suitable for spike sorting. Specifically, AEC and DeepECT consistently underperformed, even compared to the simplest traditional approach of combining PCA with K-Means. DCN’s approach of creating K-Means friendly spaces and DipDECK’s approach based on Hartigan’s Dip test appear to be more suitable for real datasets than synthetic datasets. Isomap’s competitive performance on synthetic datasets and one of the real datasets suggests that future developments in spike sorting could benefit from further exploration of manifold learning approaches. However, t-SNE and DM performed well on synthetic datasets, have obtained significantly lower performances on real datasets, even lower than PCA. This indicates that not all non-linear feature extraction methods are suitable for the spike sorting task.

The superior performance of deep clustering approaches can be attributed to their ability to learn non-linear representations (through autoencoders architectures in most approaches) that better capture the complex structure of spike data while simultaneously optimizing clustering objective. This dual optimization enables them to create feature spaces tailored for clustering which combines the feature extraction and clustering steps of spike sorting into one. Moreover, in the traditional spike sorting pipeline, because these two steps are separated, there is no guarantee that the feature extraction step provided an adequate clustering space. This again is solved through the deep clustering approach.

Our hypothesis is that the performance differences between synthetic and real datasets for certain models may stem from the inherent limitations of synthetic datasets in reproducing all aspects of in vivo recordings. While the synthetic datasets used are based on real spike shapes, they employ stationary noise models and spike shape templates that do not capture the full variability caused by biological processes (e.g., bursting patterns, synaptic modulation) or artifacts (e.g., electrode drift, overlapping spikes). The synthetic multi-unit clusters, though complex, still consist of spikes with controlled amplitude and timing distributions, which may produce more separable structures than those found in real extracellular recordings. In contrast, real data exhibit non-stationary noise, irregular cluster geometries, and non-Gaussian variability in spike shapes, which can alter algorithm performance. These differences likely explain why some methods excelled in synthetic datasets but not in real datasets, and vice versa.

### Limitations and Future Directions

Our study analyses whether deep clustering approaches could be a potential substitute for both feature extraction and clustering in traditional spike sorting pipelines. Nevertheless, limitations should be addressed in future work. Due to the computational resources required by deep clustering approaches, for real-time spike sorting optimizations or specialized hardware may be required. However, as shown by the time analysis, not all deep clustering algorithms have the same execution time, DDC has shown a high performance with an execution time comparable to that of LLE. As recording hardware advances, dataset sizes increase. Future work could evaluate the scalability of these methods. The datasets used incorporated realistic noise levels and scenarios; however, investigation could be conducted into the robustness of deep clustering methods against various noise types introduced into the neural signals and identification of drowned spikes.

Since the 1950 s, the number of recorded neurons has seen an exponential increase (Pachitariu et al., 2016). With the advancement of recording hardware (Steinmetz et al., 2021; Jun et al., 2017)that permit the simultaneous recording of thousands of neurons (Steinmetz et al., 2018), analysis methods must advance as well to deal with the increasingly large volume of data recorded. Our work is limited by single-channel data analysis. Single-channel data analysis suffers from several drawbacks in comparison to multi-channel data analysis: a lack of spatial information which increases the difficulty of attributing spikes to their source neurons (Tóth et al., 2021), overlapping spikes may not be identifiable (Rossant et al., 2016), electrode drift cannot be estimated perfectly due to the lack of spatial information (Steinmetz et al., 2021; Georgiadis et al., 2025), and a higher signal-to-noise ratio is required reducing the amount of drowned spikes that can be identified. In contrast, multi-channel data, such as those obtained from high-density probes (Steinmetz et al., 2021; Jun et al., 2017), incorporate spatial information as spikes can be identified along multiple adjacent electrodes as a multi-channel waveform which results in better separation (Jia et al., 2019; Ye et al., 2024)between spikes of different neurons and even cell type identification (Ye et al., 2024). Moreover, high-density probes through the spatial information offered allow for easier drift correction due to their coverage resulting in drift appearing as a spatial shift (Steinmetz et al., 2018). Recent research has been focused on multi-channel data analysis (Buccino et al., 2022), especially on high-density probes (Steinmetz et al., 2021; Jun et al., 2017; Pachitariu et al., 2024), yet that does not mean that the single-channel analysis has stopped (Buccino et al., 2022; Han et al., 2025; Li et al., 2020). Recent studies on neural network architectures in spike sorting as shown great promise (Cao et al., 2025) even outperforming state-of-the-art methods (Pachitariu et al., 2024). Even though our study focuses on single-channel data, it establishes a baseline on deep clustering methods and opens avenues of further research of these methods in spike sorting.

Another potential limitation of our work is the use of PCA for visualization, as it may project non-linearly separable high-dimensional embeddings into a 2D non-separable linear space. This may make clusters appear overlapped when in reality the non-linear embeddings obtained by the deep clustering methods are not. We chose PCA for consistency and interpretability across both deep and traditional methods, but we note that it may underrepresent the true separation achieved in the learned embeddings.

Purity, despite not penalizing over-clustering and tending to give higher scores for a higher number of clusters or imbalanced cluster distributions, remains a widely used clustering evaluation metric in general machine learning (Ulu & Türkan 2024). We include Purity both for comparability to previous work (not necessarily spike sorting related) and for its intuitive interpretability. However, our study does not rely on Purity alone. Instead, it is considered alongside five additional metrics to provide a robust, balanced evaluation that mitigates its known biases.

Not only the purity metric, but also the internal performance metrics have inherent limitations (Hassan et al., 2024). CHS does not work well for highly irregular shapes and can be influenced by outliers (Hassan et al., 2024), DBS is sensitive to the parameter choice of the clustering algorithm, to non-Euclidean distance metrics, and to overlapping clusters (Hassan et al., 2024), and SS may not be optimal in scenarios with noise and outliers, it requires a distance metric and for large datasets the distance matrix computation may be expensive (Hassan et al., 2024). However, SS demonstrated excellent performance in a benchmark of validity indices (Ikotun et al., 2025). Moreover, an extensive comparative study has shown that SS has the best results (Arbelaitz et al., 2013)and it indicated that some of the best performing clustering metrics are indeed SS, CHS and DBS (Arbelaitz et al., 2013). SS, DBS and CHS are still used today in the evaluation of clustering performance (Wang & Ye 2024). While, the sum of squared errors (SSE) may also be a potential clustering metric, it has been shown to have limited usefulness in non-globular clusters (Ansari et al., 2015). Nevertheless, SSE represents a potential future avenue of research. It should be noted that the inherent limitations of the existent internal clustering performance metrics may influence the interpretation of results. The fact that the best performing metrics may not yet be optimal opens another avenue of future research into clustering validity indices in the attempt to create a more robust metric.

In our analysis of synthetic datasets, we assumed that the true number of clusters *k* was known and supplied it to all clustering algorithms. Through this assumption, we have both simplified the comparison and ensured fairness in evaluating clustering performance. However, in practical spike sorting applications, the number of clusters is unknown. Many traditional clustering algorithms require *k*as an input parameter, such as K-Means, K-Medoids, and Gaussian Mixture Models. Conversely, several approaches exist for estimating the number of clusters (Mahmud et al., 2023; Bai & Chu 2025), such as Elbow method, Gap statistic, or even methods including internal validity indices like the SS, CHS, and DBS. Incorporating cluster-number estimation methods would necessitate an alternative experimental design focused on unsupervised model selection rather than controlled comparison. We consider this an important and promising direction for future research, which could build directly on the performance baselines established in our work.

While our evaluation focuses on dataset-level performance metrics, an extension of this work would be to investigate clustering performance at the level of individual cells. This analysis would be applicable to both the synthetic datasets and to the real datasets used in our work, due to the intracellular spikes which may be considered as a ground truth. Such a per-cluster (or per-cell) analysis could quantify how firing rate, spike amplitude, and cluster size influence performance. This is particularly relevant in extracellular recordings where cells with low firing rates or small amplitudes may be labelled as noise or included in larger clusters. By relating each cell’s intrinsic properties to its clustering performance, future studies could identify biases of specific algorithms.

In conclusion, our findings suggest that deep clustering algorithms, particularly DDC and VaDE, represent promising approaches for spike sorting that can overcome limitations of traditional methods. Their ability to jointly optimize feature extraction and clustering objectives makes them well-suited for the complex task of identifying individual neuronal activity in extracellular recordings. Due to the advancement of recording hardware, enabling the simultaneous recording of thousands of neurons, such advanced clustering approaches will become increasingly important for accurate spike sorting and subsequent neuroscientific discoveries.

## Supplementary Information

Below is the link to the electronic supplementary material.

Supplementary Material 1

## Data Availability

The datasets used in this work are openly available and can be found at:•Synthetic datasets (22): https://spikeforest.flatironinstitute.org/studyset/SYNTH_MONOTRODE or http://bioweb.me/CPGJNM2012-dataset or https://www.kaggle.com/datasets/ardeleanrichard/simulationsdataset/data•Real datasets (87,88): https://spikeforest.flatironinstitute.org/study/paired_kampff or https://crcns.org/data-sets/methods/spe-1/about-spe-1.
