# Supplementary material for: A Study of Deep Clustering in Spike Sorting
Source: Neuroinformatics. 2025 Oct 22;23(4):51. doi: 10.1007/s12021-025-09751-4 (PMC12546415; doi:10.1007/s12021-025-09751-4)
Supplement: Supplementary file 1 — Supplementary Material 1 [file 12021_2025_9751_MOESM1_ESM.docx]

# Supplementary Material

Table S1. A short description of each deep clustering method with its key characteristics.

| **Method** | **Embedding** | **Clustering mechanism** | **Specific mechanism** |
| --- | --- | --- | --- |
| ACeDeC | Autoencoder with separated latent spaces (cluster-specific/shared) | Centroid-based in latent space | Separates shared vs. cluster info; modified loss |
| AEC | Autoencoder latent space | Centroid-based in latent space | Combines reconstruction + clustering loss; sensitive to initialization |
| DCN | Autoencoder latent space (pretrained) | Jointly optimized K-Means | Learns “K-Means friendly” space |
| DDC | Conv. autoencoder + t-SNE reduction | Density-based clustering | Handles arbitrary shapes; depends on t-SNE properties |
| DEC | Autoencoder latent space | KL divergence to target distribution | Iterative refinement; pretraining helps |
| DeepECT | Autoencoder latent space | Hierarchical clustering tree | Builds cluster hierarchy; no cluster count needed |
| DipDECK | Autoencoder latent space | Merge clusters via Dip test | Estimates cluster number; merges based on multimodality |
| DipEncoder | Autoencoder latent space | Dip test label updates | Enforces unimodality within clusters; parameter-free |
| DKM | Autoencoder latent space | Differentiable K-Means | Continuous relaxation of K-Means objective |
| IDEC | Autoencoder latent space | KL divergence + reconstruction | Preserves local structure during clustering |
| N2D | Autoencoder + manifold learning (UMAP) | Shallow clustering | Preserves local/global structure; depends on manifold method |
| VaDE | Variational autoencoder latent space | Gaussian Mixture Model | Generative; models data distribution |

Table S2. A small subset of parameter combinations tested through grid search for the deep clustering algorithms on dataset Sim1.

| **Method** | **Pretrain params** | **Clustering params** | **Additional params** | **ARI** | **AMI** | **Purity** | **SS** | **CHS** | **DBS** |
| --- | --- | --- | --- | --- | --- | --- | --- | --- | --- |
| ACeDeC | lr=0.01 | lr=0.01 |  | 0.633 | 0.836 | 0.901 | 0.238 | 3707.013 | 1.813 |
| ACeDeC | lr=0.01 | lr=0.001 |  | 0.640 | 0.845 | 0.901 | 0.231 | 3704.399 | 1.738 |
| ACeDeC | lr=0.01 | lr=0.0001 |  | 0.478 | 0.723 | 0.759 | 0.127 | 2734.496 | 3.003 |
| ACeDeC | lr=0.001 | lr=0.01 |  | 0.541 | 0.779 | 0.840 | 0.182 | 3282.780 | 1.932 |
| ACeDeC | lr=0.001 | lr=0.001 |  | 0.659 | 0.843 | 0.870 | 0.226 | 3644.036 | 1.994 |
| ACeDeC | lr=0.001 | lr=0.0001 |  | 0.624 | 0.834 | 0.886 | 0.223 | 3669.766 | 1.696 |
| ACeDeC | lr=0.0001 | lr=0.001 |  | 0.555 | 0.785 | 0.839 | 0.193 | 3392.679 | 2.084 |
| ACeDeC | lr=0.0001 | lr=0.0001 |  | 0.569 | 0.807 | 0.846 | 0.200 | 3479.228 | 2.033 |
| DCN | lr=0.01 | lr=0.01 |  | 0.545 | 0.792 | 0.752 | 0.150 | 2846.730 | 3.247 |
| DCN | lr=0.01 | lr=0.001 |  | 0.518 | 0.756 | 0.693 | 0.164 | 2741.634 | 3.370 |
| DCN | lr=0.01 | lr=0.0001 |  | 0.480 | 0.708 | 0.672 | 0.129 | 2368.609 | 3.949 |
| DCN | lr=0.001 | lr=0.01 |  | 0.407 | 0.666 | 0.650 | 0.090 | 2095.229 | 4.180 |
| DCN | lr=0.001 | lr=0.001 |  | 0.616 | 0.797 | 0.745 | 0.179 | 2408.484 | 4.300 |
| DCN | lr=0.001 | lr=0.0001 |  | 0.608 | 0.748 | 0.711 | 0.148 | 2217.510 | 4.412 |
| DCN | lr=0.0001 | lr=0.01 |  | 0.311 | 0.557 | 0.618 | 0.088 | 2075.622 | 3.118 |
| DCN | lr=0.0001 | lr=0.001 |  | 0.530 | 0.741 | 0.756 | 0.151 | 2654.487 | 3.276 |
| DCN | lr=0.0001 | lr=0.0001 |  | 0.456 | 0.666 | 0.694 | 0.080 | 2212.639 | 3.329 |
| DDC | lr=0.01 |  | ratio=0.05 | 0.449 | 0.748 | 0.852 | 0.106 | 1796.574 | 4.299 |
| DDC | lr=0.001 |  | ratio=0.05 | 0.488 | 0.766 | 0.848 | 0.148 | 2484.949 | 2.788 |
| DDC | lr=0.01 |  | ratio=0.1 | 0.551 | 0.813 | 0.727 | 0.209 | 3830.586 | 1.609 |
| DDC | lr=0.001 |  | ratio=0.1 | 0.643 | 0.828 | 0.754 | 0.266 | 4130.306 | 1.091 |
| DDC | lr=0.01 |  | ratio=0.15 | 0.526 | 0.767 | 0.616 | 0.288 | 4995.461 | 1.164 |
| DDC | lr=0.001 |  | ratio=0.15 | 0.546 | 0.770 | 0.651 | 0.287 | 4331.491 | 1.146 |
| IDEC | lr=0.01 | lr=0.001 | alpha=0.1 | 0.760 | 0.863 | 0.768 | 0.128 | 2604.130 | 2.612 |
| IDEC | lr=0.01 | lr=0.0001 | alpha=0.1 | 0.686 | 0.808 | 0.748 | 0.157 | 2512.591 | 2.867 |
| IDEC | lr=0.01 | lr=1e-05 | alpha=0.1 | 0.730 | 0.831 | 0.828 | 0.193 | 3119.466 | 2.443 |
| IDEC | lr=0.001 | lr=0.001 | alpha=0.1 | 0.703 | 0.831 | 0.721 | 0.131 | 2289.694 | 3.219 |
| IDEC | lr=0.001 | lr=0.0001 | alpha=0.1 | 0.579 | 0.800 | 0.721 | 0.130 | 2369.508 | 3.016 |
| IDEC | lr=0.001 | lr=1e-05 | alpha=0.1 | 0.615 | 0.774 | 0.731 | 0.136 | 2622.023 | 3.126 |
| IDEC | lr=0.0001 | lr=0.001 | alpha=0.1 | 0.361 | 0.574 | 0.452 | 0.058 | 843.055 | 7.229 |
| IDEC | lr=0.0001 | lr=0.0001 | alpha=0.1 | 0.439 | 0.683 | 0.511 | 0.025 | 1198.850 | 5.224 |
| IDEC | lr=0.0001 | lr=1e-05 | alpha=0.1 | 0.598 | 0.664 | 0.633 | 0.051 | 1739.837 | 3.689 |
| IDEC | lr=1e-05 | lr=0.001 | alpha=0.1 | 0.592 | 0.712 | 0.607 | 0.147 | 1953.468 | 6.178 |
| IDEC | lr=1e-05 | lr=0.0001 | alpha=0.1 | 0.523 | 0.705 | 0.679 | 0.090 | 2049.823 | 4.571 |
| IDEC | lr=1e-05 | lr=1e-05 | alpha=0.1 | 0.334 | 0.570 | 0.586 | 0.002 | 1362.409 | 6.037 |
| IDEC | lr=0.01 | lr=0.001 | alpha=0.25 | 0.698 | 0.832 | 0.717 | 0.106 | 2379.412 | 2.945 |
| IDEC | lr=0.01 | lr=0.0001 | alpha=0.25 | 0.696 | 0.791 | 0.759 | 0.144 | 2458.774 | 3.493 |
| IDEC | lr=0.01 | lr=1e-05 | alpha=0.25 | 0.632 | 0.813 | 0.827 | 0.195 | 3368.741 | 1.973 |
| IDEC | lr=0.001 | lr=0.001 | alpha=0.25 | 0.789 | 0.876 | 0.776 | 0.150 | 2559.573 | 2.775 |
| IDEC | lr=0.001 | lr=0.0001 | alpha=0.25 | 0.793 | 0.855 | 0.782 | 0.152 | 2660.362 | 3.217 |
| IDEC | lr=0.001 | lr=1e-05 | alpha=0.25 | 0.600 | 0.772 | 0.762 | 0.138 | 2756.745 | 2.888 |
| IDEC | lr=0.0001 | lr=0.001 | alpha=0.25 | 0.406 | 0.640 | 0.499 | 0.064 | 1332.111 | 5.655 |
| IDEC | lr=0.0001 | lr=0.0001 | alpha=0.25 | 0.515 | 0.746 | 0.653 | 0.101 | 1728.924 | 4.869 |
| IDEC | lr=0.0001 | lr=1e-05 | alpha=0.25 | 0.512 | 0.655 | 0.629 | 0.036 | 1826.551 | 3.574 |
| IDEC | lr=1e-05 | lr=0.001 | alpha=0.25 | 0.474 | 0.651 | 0.525 | 0.040 | 1529.274 | 6.699 |
| IDEC | lr=1e-05 | lr=0.0001 | alpha=0.25 | 0.673 | 0.767 | 0.688 | 0.115 | 2111.379 | 4.311 |
| IDEC | lr=1e-05 | lr=1e-05 | alpha=0.25 | 0.329 | 0.540 | 0.582 | 0.006 | 1470.610 | 5.719 |
| IDEC | lr=0.01 | lr=0.001 | alpha=0.5 | 0.735 | 0.833 | 0.757 | 0.084 | 2426.251 | 2.478 |
| IDEC | lr=0.01 | lr=0.0001 | alpha=0.5 | 0.664 | 0.788 | 0.746 | 0.113 | 2428.083 | 3.868 |
| IDEC | lr=0.01 | lr=1e-05 | alpha=0.5 | 0.831 | 0.838 | 0.859 | 0.218 | 3191.731 | 1.922 |
| IDEC | lr=0.001 | lr=0.001 | alpha=0.5 | 0.846 | 0.895 | 0.835 | 0.185 | 3022.735 | 2.735 |
| IDEC | lr=0.001 | lr=0.0001 | alpha=0.5 | 0.793 | 0.859 | 0.783 | 0.166 | 2656.840 | 3.642 |
| IDEC | lr=0.001 | lr=1e-05 | alpha=0.5 | 0.607 | 0.771 | 0.781 | 0.142 | 2786.063 | 2.990 |
| IDEC | lr=0.0001 | lr=0.001 | alpha=0.5 | 0.319 | 0.645 | 0.484 | -0.016 | 1332.271 | 4.141 |
| IDEC | lr=0.0001 | lr=0.0001 | alpha=0.5 | 0.528 | 0.747 | 0.658 | 0.084 | 1760.022 | 5.090 |
| IDEC | lr=0.0001 | lr=1e-05 | alpha=0.5 | 0.421 | 0.650 | 0.627 | 0.024 | 1849.134 | 3.896 |
| IDEC | lr=1e-05 | lr=0.001 | alpha=0.5 | 0.562 | 0.726 | 0.647 | 0.074 | 1474.762 | 5.779 |
| IDEC | lr=1e-05 | lr=0.0001 | alpha=0.5 | 0.460 | 0.701 | 0.641 | 0.082 | 2194.190 | 4.814 |
| IDEC | lr=1e-05 | lr=1e-05 | alpha=0.5 | 0.550 | 0.580 | 0.610 | 0.040 | 1544.801 | 6.493 |
| IDEC | lr=0.01 | lr=0.001 | alpha=0.75 | 0.605 | 0.816 | 0.722 | 0.105 | 2567.319 | 3.497 |
| IDEC | lr=0.01 | lr=0.0001 | alpha=0.75 | 0.655 | 0.796 | 0.733 | 0.133 | 2390.823 | 3.318 |
| IDEC | lr=0.01 | lr=1e-05 | alpha=0.75 | 0.695 | 0.788 | 0.784 | 0.172 | 2737.930 | 2.463 |
| IDEC | lr=0.001 | lr=0.001 | alpha=0.75 | 0.784 | 0.873 | 0.820 | 0.125 | 3017.833 | 2.267 |
| IDEC | lr=0.001 | lr=0.0001 | alpha=0.75 | 0.795 | 0.859 | 0.782 | 0.183 | 2656.586 | 3.293 |
| IDEC | lr=0.001 | lr=1e-05 | alpha=0.75 | 0.630 | 0.765 | 0.747 | 0.135 | 2601.481 | 3.565 |
| IDEC | lr=0.0001 | lr=0.001 | alpha=0.75 | 0.405 | 0.669 | 0.552 | 0.010 | 1426.989 | 4.698 |
| IDEC | lr=0.0001 | lr=0.0001 | alpha=0.75 | 0.446 | 0.743 | 0.652 | 0.087 | 1913.730 | 3.983 |
| IDEC | lr=0.0001 | lr=1e-05 | alpha=0.75 | 0.386 | 0.660 | 0.628 | 0.050 | 1877.326 | 3.785 |
| IDEC | lr=1e-05 | lr=0.001 | alpha=0.75 | 0.504 | 0.681 | 0.616 | 0.047 | 1587.706 | 6.190 |
| IDEC | lr=1e-05 | lr=0.0001 | alpha=0.75 | 0.369 | 0.635 | 0.571 | 0.062 | 1424.059 | 5.671 |
| IDEC | lr=1e-05 | lr=1e-05 | alpha=0.75 | 0.433 | 0.659 | 0.687 | 0.100 | 2285.574 | 3.657 |
| VaDE | lr=0.01 | lr=0.01 |  | 0.594 | 0.744 | 0.630 | 0.148 | 1777.147 | 1.971 |
| VaDE | lr=0.01 | lr=0.001 |  | 0.826 | 0.877 | 0.855 | 0.125 | 1301.421 | 2.377 |
| VaDE | lr=0.01 | lr=0.0001 |  | 0.597 | 0.827 | 0.803 | 0.103 | 1223.852 | 2.827 |
| VaDE | lr=0.001 | lr=0.01 |  | 0.191 | 0.387 | 0.328 | 0.163 | 2792.460 | 1.972 |
| VaDE | lr=0.001 | lr=0.001 |  | 0.704 | 0.886 | 0.918 | 0.123 | 1269.372 | 2.881 |
| VaDE | lr=0.001 | lr=0.0001 |  | 0.617 | 0.842 | 0.871 | 0.116 | 1273.321 | 2.702 |
| VaDE | lr=0.0001 | lr=0.01 |  | 0.521 | 0.763 | 0.615 | 0.181 | 2311.056 | 1.765 |
| VaDE | lr=0.0001 | lr=0.001 |  | 0.693 | 0.875 | 0.895 | 0.126 | 1287.127 | 2.332 |
| VaDE | lr=0.0001 | lr=0.0001 |  | 0.731 | 0.891 | 0.911 | 0.131 | 1353.066 | 2.539 |


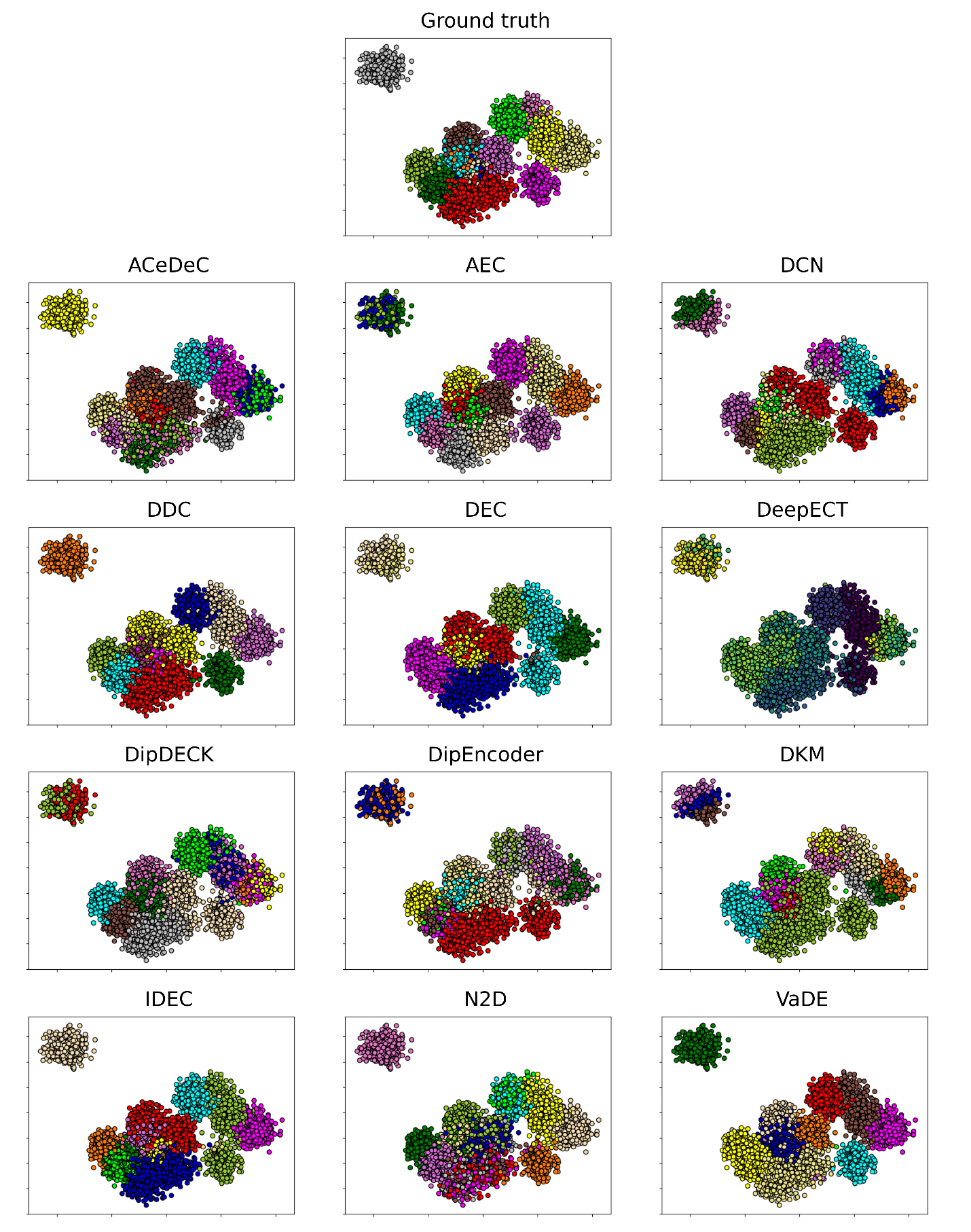


Fig S1. Deep clustering methods applied on the Sim20 dataset. Colors represent the clustering labels obtained in the same PCA 2d project space. The multi-unit cluster is colored red for the ground truth scatter plot and it has the same localization in each plot.

Table S3. Analysis of performance of algorithms for the Sim2 dataset in a one-vs-rest label setup with one single-unit cluster.

| **Algorithm** | **ARI** | **AMI** | **Purity** | **SS** | **CHS** | **DBS** |
| --- | --- | --- | --- | --- | --- | --- |
| PCA | 0.002 | 0.039 | 0.978 | 0.294 | 6603.085 | 1.316 |
| ICA | 0.002 | 0.039 | 0.978 | 0.294 | 6602.936 | 1.316 |
| Isomap | 0.000 | 0.015 | 0.978 | 0.292 | 6521.006 | 1.321 |
| LLE | -0.025 | 0.005 | 0.978 | 0.288 | 1141.357 | 0.827 |
| t-SNE | -0.002 | 0.036 | 0.978 | 0.246 | 4934.372 | 1.492 |
| DM | -0.025 | 0.005 | 0.978 | 0.288 | 1141.357 | 0.827 |
| ACeDeC | 0.038 | 0.061 | 0.978 | 0.226 | 4004.625 | 1.628 |
| AEC | 0.065 | 0.075 | 0.978 | 0.274 | 4557.591 | 1.477 |
| DCN | -0.031 | 0.014 | 0.978 | 0.301 | 3593.800 | 1.326 |
| DDC | 0.008 | 0.057 | 0.978 | 0.277 | 3813.128 | 1.287 |
| DEC | 0.026 | 0.054 | 0.978 | 0.217 | 3814.702 | 1.693 |
| DKM | -0.015 | 0.003 | 0.978 | 0.307 | 4060.977 | 1.477 |
| DeepECT | 0.002 | 0.026 | 0.978 | 0.008 | 1140.477 | 3.819 |
| DipDECK | 0.002 | 0.033 | 0.978 | 0.248 | 4060.397 | 1.561 |
| DipEncoder | 0.029 | 0.049 | 0.978 | 0.201 | 3511.682 | 1.755 |
| IDEC | 0.004 | 0.040 | 0.978 | 0.292 | 6542.057 | 1.325 |
| N2D | 0.007 | 0.040 | 0.978 | 0.152 | 1862.105 | 2.459 |
| VaDE | -0.020 | 0.024 | 0.978 | 0.233 | 4935.803 | 1.328 |

Table S4. Analysis of performance for varying number of clusters (*k*) as input to the IDEC algorithm.

| **k** | **ARI** | **AMI** | **Purity** | **SS** | **CHS** | **DBS** |
| --- | --- | --- | --- | --- | --- | --- |
| 2 | 0.004 | 0.040 | 0.978 | 0.292 | 6542.057 | 1.325 |
| 4 | 0.034 | 0.112 | 0.914 | 0.215 | 4076.964 | 1.699 |
| 6 | 0.142 | 0.370 | 0.903 | 0.231 | 3698.307 | 1.792 |
| 8 | 0.145 | 0.410 | 0.771 | 0.202 | 3082.434 | 2.513 |
| 10 | 0.137 | 0.467 | 0.744 | 0.215 | 3135.471 | 2.365 |
| 12 | 0.196 | 0.560 | 0.762 | 0.217 | 2997.071 | 2.160 |
| 14 | 0.371 | 0.698 | 0.774 | 0.211 | 2789.036 | 2.461 |
| 16 | 0.500 | 0.769 | 0.818 | 0.205 | 2653.404 | 2.693 |
| 18 | 0.552 | 0.801 | 0.838 | 0.199 | 2523.571 | 3.173 |
| 20 | 0.855 | 0.879 | 0.837 | 0.189 | 2410.605 | 3.402 |


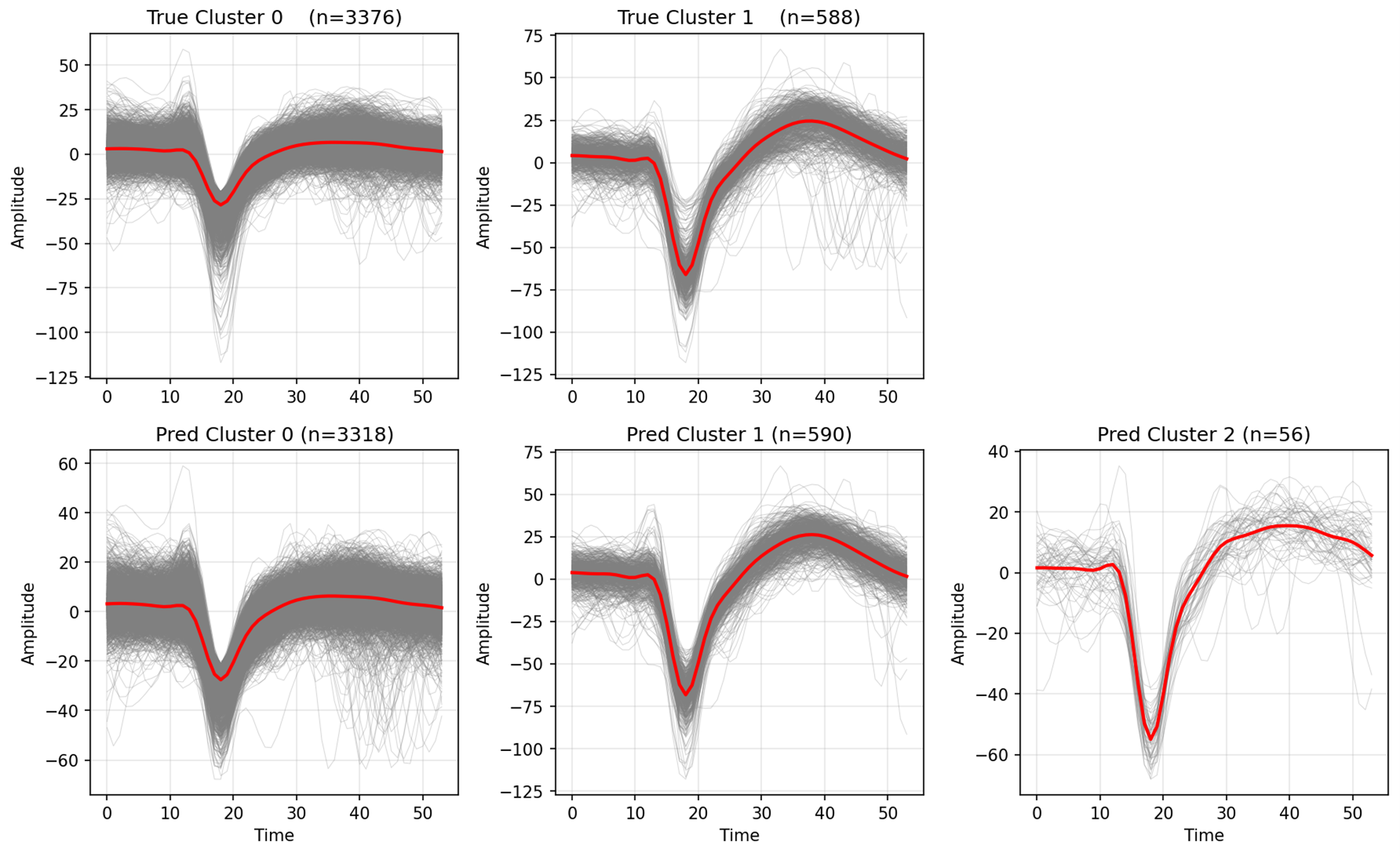


Figure S2. The distribution of true and predicted clusters as signals (red line represents the mean) for the DCN algorithm on the c37 real dataset (true cluster 1 represents the intracellular spikes)


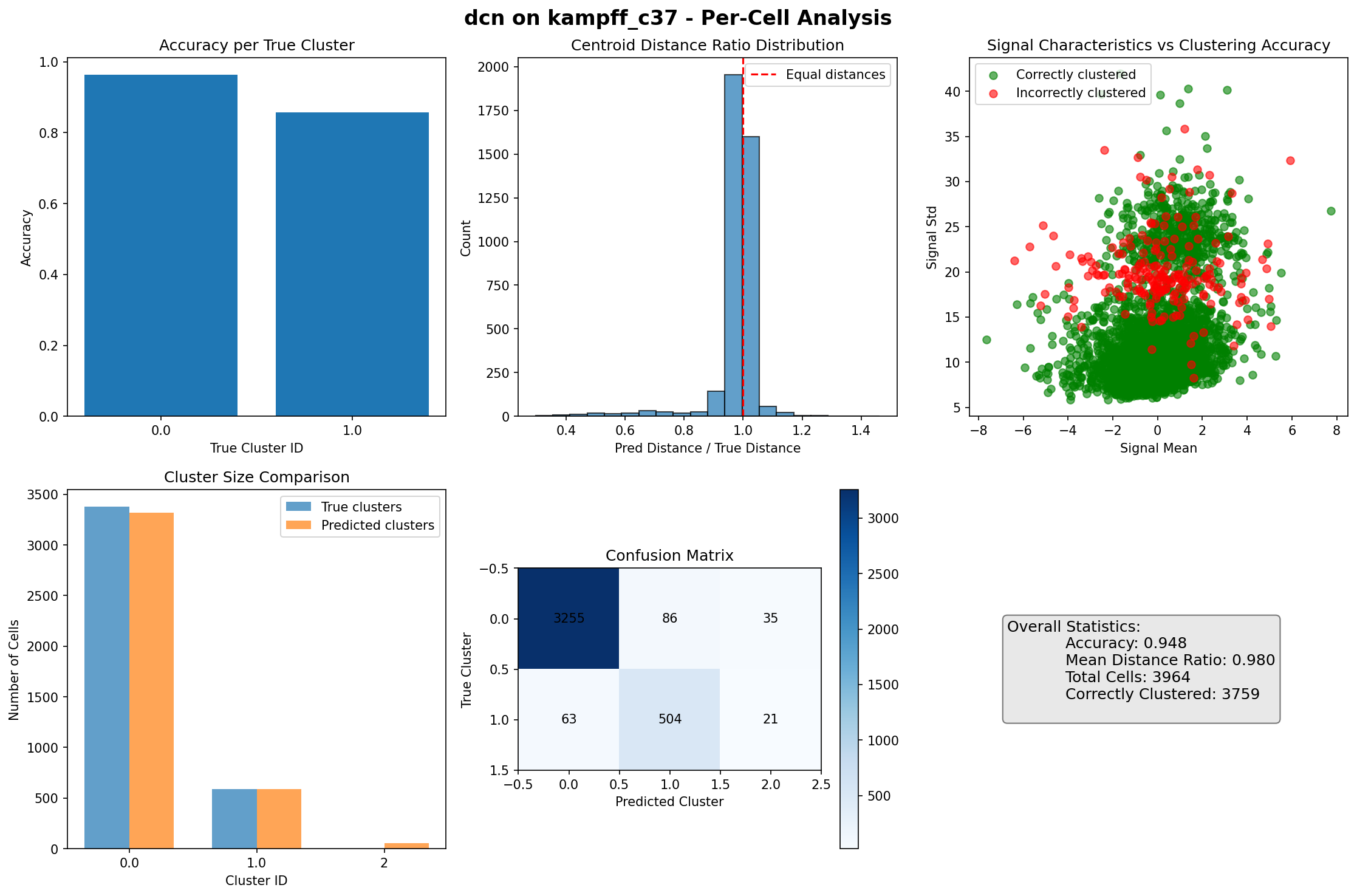


Fig S3. Statistics of the result obtained by the DCN algorithm on the c37 real dataset.
